# Family dinner: Transcriptional plasticity of five Noctuidae (Lepidoptera) feeding on three host plant species

**DOI:** 10.1002/ece3.9258

**Published:** 2022-09-06

**Authors:** Thijmen Breeschoten, M. Eric Schranz, Erik H. Poelman, Sabrina Simon

**Affiliations:** ^1^ Biosystematics Group Wageningen University & Research Wageningen The Netherlands; ^2^ Laboratory of Entomology Wageningen University & Research Wageningen The Netherlands

**Keywords:** detoxification genes, generalists, phylogenetic framework, polyphagy, RNA‐seq, transcriptomics

## Abstract

Polyphagous insects often show specialization in feeding on different host plants in terms of survival and growth and, therefore, can be considered minor or major pests of particular hosts. Whether polyphagous insects employ a common transcriptional response to cope with defenses from diverse host plants is under‐studied. We focused on patterns of transcriptional plasticity in polyphagous moths (Noctuidae), of which many species are notorious pests, in relation to herbivore performance on different host plants. We compared the transcriptional plasticity of five polyphagous moth species feeding and developing on three different host plant species. Using a comparative phylogenetic framework, we evaluated if successful herbivory, as measured by larval performance, is determined by a shared or lineage‐specific transcriptional response. The upregulated transcriptional activity, or gene expression pattern, of larvae feeding on the different host plants and artificial control diet was highly plastic and moth species‐specific. Specialization, defined as high herbivore success for specific host plants, was not generally linked to a lower number of induced genes. Moths that were more distantly related and showing high herbivore success for certain host plants showed shared expression of multiple homologous genes, indicating convergence. We further observed specific transcriptional responses within phylogenetic lineages. These expression patterns for specific host plant species are likely caused by shared evolutionary histories, for example, symplesiomorphic patterns, and could therefore not be associated with herbivore success alone. Multiple gene families, with roles in plant digestion and detoxification, were widely expressed in response to host plant feeding but again showed highly moth species‐specific. Consequently, high herbivore success for specific host plants is also driven by species‐specific transcriptional plasticity. Thus, potential pest moths display a complex and species‐specific transcriptional plasticity.

## Graphical abstract

Cutworm moths (Noctuidae) are polyphagous species feeding on a large number of plant species and some are considered notorious pests. In our study, we performed feeding essays of five polyphagous moth species feeding and developing on three different host plant species and linked their herbivore success to their transcriptional response based on newly generated expression (Illumina RNA‐seq) data. Successful polyphagous moths have a shared transcriptional response but rely largely on a species‐specific transcriptional plasticity.

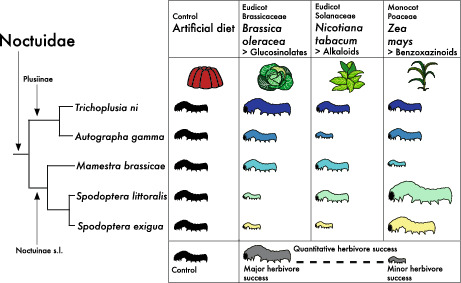

## 1 INTRODUCTION

Herbivorous insects and their host plants have evolved various forms of specialization, often forming the foundation of ecological and evolutionary theories (Funk et al., 2002; Kawecki, 1998). Host specialization among herbivorous insects is common. Specialization is often seen as a consequence of the co‐evolution of plant defenses and herbivore resistance, resulting in both species and gene evolution (Edger et al., 2015; Ehrlich & Raven, 1964; Heidel‐Fischer & Vogel, 2015). Plants defend themselves from herbivores by both physical barriers and by synthesizing toxins (allelochemicals/specialized metabolites) (Després et al., 2007; War et al., 2018). Despite these defenses, many herbivorous insects are polyphagous and have maintained and/or evolved the ability to feed on a broad set of host plants, even including multiple plant families (Bernays & Graham, 1988; Schoonhoven et al., 2005).

A general molecular mechanism for herbivores to cope with plant chemical defenses is through detoxification (Després et al., 2007). Various major gene families, well known to be involved in the three‐phased detoxification pathway (Kant et al., 2015), are generally conserved across insects. In the first phase, gene families including cytochrome P450 monooxygenases (P450s), oxidoreductases, and carboxyl‐ and choline esterases (CCEs) metabolize the toxin (Després et al., 2007; Heckel, 2018; Li et al., 2007; Müller et al., 2017; Schuler, 2011; Zhang et al., 2013). In the second phase, they are conjugated by families including UDP‐glycosyltransferases (UGTs) and glutathione S‐transferases (GSTs) before they, in the third phase, get transported away by transporters including ATP‐binding cassettes (ABCs) (Bock, 2016; Francis et al., 2005; Heidel‐Fischer & Vogel, 2015; Kant et al., 2015).

Detoxification is not restricted to these well‐known gene families. Indeed, various insect species, often specializing on narrow groups of plant species, have evolved specialized resistance methods differing from those described above, such as the nitrile‐specifier protein (NSP) in Pierinae (Fischer et al., 2008; Wheat et al., 2007; Wittstock et al., 2004) and glucosinolate sulfatase (GSS) in the diamondback moth (*Plutella xylostella*) (Heidel‐Fischer et al., 2019; Ratzka et al., 2002). The detoxification and metabolism of plant defense toxins is most likely due to concerted functioning of genes from these various shared and specific gene families and is expected to vary with different toxins.

A polyphagous herbivore requires plasticity in its transcriptional response to overcome the diversity in plant defenses (Orsucci et al., 2018). Transcriptional changes in genes involved in xenobiotic detoxification and digestion especially play a central role when facing different host plant species (Celorio‐Mancera et al., 2012; Celorio‐Mancera et al., 2013; Dermauw, Wybouw, et al., 2013; Rivard et al., 2004; Wybouw et al., 2015). For example, in the green peach aphid (*Myzus persicae*) transcriptional plasticity facilitates the shift to a new host plant by differential regulation of specific gene clusters (Mathers et al., 2017). Moreover, in aphids the co‐regulated expression of effector genes was suggested to form a transcriptional mechanism enabling plant feeding (Thorpe et al., 2018). Transcriptional plasticity has also been demonstrated in the beet armyworm (*Spodoptera exigua*), where specific upregulated expression clusters are observed in moths feeding on host plant species, which are sub‐optimal (Breeschoten et al., 2019). It has been found that Lepidoptera species feeding on a wide host plant range display broad plasticity in transcriptional responses (Celorio‐Mancera et al., 2013; Pearce et al., 2017; Schweizer et al., 2017). However, whether a general transcriptional plastic pattern in response to host plant feeding exists across polyphagous insects is largely unknown.

The existence of a shared transcriptional response among polyphagous insects exhibiting successful herbivory is of importance to understand and anticipate on polyphagous insects forming pests (pest formations). Within polyphagous insect species, populations can be restricted in their host plant choice in terms of successful feeding and development (Howard et al., 1994; Pashley, 1986; Schoonhoven et al., 2005); thus, a degree of specialization occurs. This “host‐specialization” could eventually lead to pest formations in certain insect species (Gouin et al., 2017).

In order to elucidate a potential shared transcriptional pattern that differentiates pest formations from unsuccessful herbivory, multi‐species comparisons need to be conducted. For the multi‐species comparisons, the polyphagous target species should display variable levels of herbivore success. It is also of importance to account for the evolutionary relationships of the target species and consequently perform comparisons through a phylogenetic framework. Data gathered from multiple species are not independent and will be affected by evolutionary relatedness. If evolutionary relatedness is not considered, the statistical assumption of independence is violated, and shared traits, such as responsiveness or as presented here transcriptional expression, are wrongly interpreted (Dunn et al., 2013; Dunn et al., 2018). Further, the inclusion of multiple species is of importance to test for general patterns and to avoid the classical two‐species comparison pitfall because any two species differ (Ali & Agrawal, 2012). By increasing the number of species, patterns of transcriptional responses and evolutionary histories, such as convergence and symplesiomorphy, become visible and evident.

In this study, we implement a comparative phylogenetic framework in order to study the transcriptional mechanisms of (un)successful herbivory, as measured by larval growth performance, in polyphagous moth species. Our experimental system included five species, the cabbage looper (*Trichoplusia ni*), the silver‐Y moth (*Autographa gamma*), the cabbage moth (*Mamestra brassicae*), the African cotton leafworm (*Spodoptera littoralis*), and the beet armyworm (*Spodoptera exigua*) (Breeschoten et al., 2019) across three of their recorded host plant species: cabbage (*Brassica oleracea*), maize (*Zea mays*), and tobacco (*Nicotiana tabacum*) (EPPO, 2019). Control diets were implemented by rearing these insects on an artificial diet, free of plant allelochemicals. The selected host plant species are members of different families that are known to employ different defense mechanisms including specialized metabolites: benzoxazinoids in *Z. mays* (Frey et al., 2009), glucosinolates in *B. oleracea* (Halkier & Gershenzon, 2006), and various alkaloids such as nicotine in *N. tabacum* (Baldwin, 1988). All included moth species belong to the cutworm moth family Noctuidae, which is a cosmopolitan species‐rich family including ~1089 genera and ~11,772 species (van Nieukerken et al., 2011). Many species in the family are major polyphagous species and known to form notorious pests, which has led authors to designate part of this family as a “pest‐clade” (Mitchell et al., 2006). Especially in the genus *Spodoptera*, major polyphagous species occur, which cause infestations in many parts of the world that destroy harvests and diminish crop yields (e.g., EPPO, 2019; Goergen et al., 2016; Kalleshwaraswamy et al., 2018).

In our comparative phylogenetic framework, we first analyzed larval performance by feeding assays to quantify herbivore success on the different plant species. We then analyzed and compared the transcriptional plasticity using RNA sequencing of feeding larvae in relation to their herbivore success. We focused on transcriptional response differences between the Noctuidae moths on different host plant species, which could be correlated with high herbivore success and thus pest potential.

## 2 MATERIALS AND METHODS

### 2.1 Noctuidae species selection, rearing, and host plant selection

Species used in the experimental feeding assays were obtained from various stock rearings. The parental generation of all Noctuidae specimens used in the feeding assays and RNA sequencing were raised on the same artificial diet at Unifarm, WUR greenhouse facilities, including *S. exigua* reported in a previous study (Breeschoten et al., 2019). The subsequent offspring generation was used in the feeding assays and RNA sequencing as presented here.

The specimens used for the *Spodoptera littoralis* originated from Andermatt Biocontrol, Grossdietwil, Switzerland. Specimens of *Trichoplusia ni* originated from Great Lakes Forestry Centre, Ontario, Canada. Both *Autographa gamma* and *Mamestra brassicae* originated from the Laboratory of Entomology, Wageningen University & Research (WUR), the Netherlands. Rearing and further experiments were done in temperature and light‐controlled greenhouse compartments at WUR (*S. littoralis*: 26°C/16:8 h light : dark photoperiod/50% humidity; *A. gamma*, *M. brassicae*, and *T. ni*: 18°C:20°C minimum night: day temperature/16:8 light : dark photoperiod). We additionally used the data from *Spodoptera exigua* reported in Breeschoten et al. (2019), for which specimens originated from the stock rearing of the Laboratory of Virology, Wageningen University & Research. Rearing was similar for all five species. Eggs were received and placed in plastic containers. Larvae were kept in these containers providing only artificial diet, consisting of water, corn flour, agar, yeast, wheat germ, sorbic acid, methylparaben, ascorbic acid, and streptomycin sulfate. The food contained seed‐derived products but is heavily processed and thus does not contain bioactive compounds present in live plant tissues consumed by larvae in the feeding assays. Larvae were transferred to vermiculite‐filled containers for pupating. Adult moths were kept in cylindrical containers; eggs were deposited on paper sheets that were placed in the containers. For *M. brassicae*, adult moths were kept in cylindrical containers covered with paper sheets for horizontal egg deposition, while *T. ni* moths primarily deposited their eggs on gauze fabric placed inside the containers. Respective egg clutches were collected and finally used for the feeding assays (see below).

Three different plant species were used in the feeding assays: *Zea mays* L. (accession B73, PI550473 ‐lot 94ncai02; propagated by self‐pollination at the University of Amsterdam, seeds provided by Dr M.E. Stam), *Brassica oleracea var. gemmifera* L. cultivar Cyrus (provided by Unifarm Wageningen University & Research, seeds from Syngenta Seeds, The Netherlands), and *Nicotiana tabacum* L. (accession TC325, PI552514; provided by Dr. J.M. Nifong, US Nicotiana Germplasm Collection). The plant growth was similar as in Breeschoten et al. (2019). Plants were sown and grown under optimal species‐specific conditions at the Unifarm Wageningen University & Research greenhouse facilities until use in the feeding assays. All plants within the experimental framework are representatives of plant families within the accepted host range of the moth species, as tested by the feeding assays.

### 2.2 Larval performance and collection for RNA‐seq

Larval performance, as an indicator for herbivore success, was quantified in feeding assays. Larvae developed and fed on a single host plant within separate breeding cages. The control group was kept on artificial diet in a plastic container. The plants used for the feeding assays were ~5–9 weeks old and still undergoing vegetative growth (e.g., not yet flowering). Multiple egg clutches, totaling ~300–400 eggs, were selected and placed on top of the leaves of the three host plants or inside the plastic container for the control group (artificial diet) setup. The larvae hatched and were allowed to develop and feed from the host plants or artificial diet until reaching the third larval stage.

A subgroup of the larvae reaching the third larval stage were used for RNA sequencing. Larvae used for RNA sequencing were collected on the first day of reaching the third larval stage, ground and frozen in RNAlater reagent (QIAGEN, Hilden, Germany) and kept at −80°C until RNA isolation. For each species, three biological replicates of five larvae each were sampled for each treatment.

Surviving larvae, not used for sequencing and to a maximum of about 100 individuals, were used for larval performance measurements. Upon reaching the third larval stage, larvae were collected on ice, frozen, and weighed within a single day on a Sartorius MSE3.6P‐000‐DM Cubis Micro Balance (Sartorius, Göttingen, Germany). Developmental time (period from hatching until reaching third larval stage, in days) was recorded to calculate the growth rate in mg per day (mg/day).

Due to previously observed high larval mortality rates in the whole‐plant setup of *N. tabacum*, a slightly modified design was adopted for all specimens, as outlined in Breeschoten et al. (2019). In brief, eggs were placed on detached leaves of *N. tabacum* in a plastic container. After hatching, leaves were regularly refreshed until larvae reached the late‐second larval stage. Afterward, larvae were transferred back to the whole‐plant setup, continuing their development and collected after 48 h. Only larvae molted to the third larval stage and with clear feeding marks (i.e., visible feeding damage) were collected.

### 2.3 Feeding assay statistics

Weight and growth rates for all diet treatments and species were checked for significance using a Kruskal–Wallis test followed by a Dunn test for pairwise comparisons using the Bonferroni method for *p*‐value adjustment in R v.3.4.3 (R Development Core Team, 2020).

For comparison of growth rates on the various host plants, the growth rates of each species were normalized using the growth rates of the respective control group. We assumed the artificial diet to be an optimal food source lacking any form of defense toxins. For normalization, we calculated the average growth rate of larvae feeding on the artificial diet (=“neutral” growth rate). Second, for every host plant, the growth rates were calculated by using the deviation in percentage from the respective species‐specific average “neutral” growth rate.

### 2.4 RNA sequencing & assembly

RNA extraction, cDNA library preparation, and sequencing were conducted by Novogene (Novogene Co., Ltd.) and are described in detail by Breeschoten et al. (2019). In brief, RNA was extracted using TRIzol Reagent (Invitrogen Co. Ltd) and the NEBNext Ultra RNA Library Prep Kit for Illumina sequencing (NEB) was used for cDNA library preparation following the manufacturer's protocol. RNA concentrations are given in Table S1, indicating the quality and consistency of RNA samples used. All File S23 were uploaded to the 4TU Centre for Research Data repository and available online: DOI:10.4121/14115386. Library quality was evaluated on an Agilent Bioanalyzer 2100 (Agilent Technologies), and libraries were sequenced on an Illumina HiSeq 4000 platform with 150 bp paired‐end (PE) reads. In total, 48 RNA libraries were sequenced including three replicates, consisting of five larvae, for all treatments.

The transcriptome assembly procedure and settings were described in detail in Breeschoten et al. (2019). In brief, quality checks of sequencing reads were done using FastQC v.0.10.1 (Andrews, 2010); filtering and adapter trimming were done using Trimmomatic v.0.36 (Bolger et al., 2014). Additionally, raw reads were checked for remaining adapter sequences and potential contamination using a local BLASTN search (NCBI‐BLAST+ v.2.6.0 [Camacho et al., 2009]) against the UniVec database (ftp.ncbi.nlm.nih.gov/pub/UniVec/, accessed: 01 February 2018 Built: 10.0). Trinity v.2.5.1 (Grabherr et al., 2011) was used for de novo transcriptome assembly. Raw reads were submitted to the NCBI SRA database under accession numbers PRJNA512462 (*S. littoralis*), PRJNA556816 (*A. gamma*), PRJNA543970 (*M. brassicae*), and PRJNA548256 (*T. ni*). Each assembled transcriptome was checked for contaminated sequences using DeconSeq v.0.4.3 (Schmieder & Edwards, 2011), and subsequently suspicious transcripts were removed. All reference transcriptomes were submitted to the NCBI TSA database: GHFD00000000 (*S. littoralis*; described here: GHFD01000000), GHUD00000000 (*A. gamma*; described here: GHUD01000000), GHNQ00000000 (*M. brassicae*; described here: GHNQ01000000), and GHOK00000000 (*T. ni*; described here: GHOK01000000). All raw sequence reads for *S. exigua* can be found under NCBI BioProject PRJNA477295 (Breeschoten et al., 2019). The transcriptome under accession GGRZ00000000, version GGRZ01000000, was used here. See Table S2 for an overview of the number of raw reads per library and the number of reads after trimming, cleaning, and contamination checks, as well as number of contigs per transcriptome before and after contamination. Completeness of the final reference transcriptome assemblies was accessed using BUSCO v.3.0.2. using the *Insecta_odb9* set for comparison (Simão et al., 2015; Table S3).

### 2.5 Expression quantification

Transcript expression analysis was done for each species separately. Reads from all sample treatments per species were mapped to the respective reference transcriptome using Bowtie v.2.3.4 (Langmead & Salzberg, 2012). Isoform and gene abundance estimates were calculated using RSEM v.1.3.0 (Li & Dewey, 2011). The per‐treatment count matrix was fed into a perl‐based script available in the Trinity package (*abundance_estimates_to_matrix.pl*) to produce a non‐normalized count matrix per species and summarizing all treatments. The species‐specific count matrix was used for the individual differential expression analyses. Differential expression analysis for each species was done with DESeq2 v.1.18.1 (Love et al., 2014) implemented in Trinity using default settings (min_rowSum_counts of 2 and considering sequencing depth and RNA composition by using the median of ratios method for normalization).

We tested for each species the sample and biological replicate relationships using principal components analysis (PCA) in the R package *stats* v.3.4.3 (R Development Core Team, 2020). We used a filtered CPM‐TMM log2 transformed and centered dataset (Figure [Link], [Link], [Link], [Link]). Centering is needed to focus on differences in expression rather than general physiological and metabolic functions. The raw count matrices were filtered on abundance and normalized using count‐per‐million values (CPM; accounting for library size differences between samples) using edgeR v.3.20.8 (Robinson et al., 2010) in R v.3.4.3 (R Development Core Team, 2020) and trimmed mean of M values (TMM; cross‐sample normalization) (Robinson & Oshlack, 2010) using “calcNormFactors” in edgeR v.3.20.8. Only genes with a minimum of 10 counts in at least two samples were considered expressed and retained for further analysis.

Finally, we extracted and clustered the differentially expressed transcripts according to their patterns of expression across the samples. We considered transcripts significantly differentially expressed with a minimal fold change (FC) of four between any of the treatments and a false discovery rate (FDR) of *p*‐value ≤1e‐3. The FC and FDR were set after initial tests. Lowering the thresholds resulted in an increase of “noise,” non‐host plant‐specific patterns, while remaining stable at more stringent values (see also Breeschoten et al. (2019)). Hierarchical clustering of the CPM and TMM normalized expression values of all differentially expressed transcripts was done using the Trinity script (*define_clusters_by_cutting_tree.pl*). The resulting dendrogram was pruned at 50% to define clusters with similar expression patterns.

### 2.6 Transcript annotation

Different strategies were applied for transcript annotation. All isoforms of the species‐specific de novo transcriptomes were annotated using the Trinotate pipeline v.3.0 (Haas et al., 2013). First, candidate coding regions were identified using Transdecoder v.5.0.2 from the Trinity package (Haas et al., 2013). A default length of minimal 100 amino acids (aa) for open reading frames (ORFs) was used. A BLASTP and BLASTX search was conducted, using either the translated protein sequences or the nucleotide‐coded transcripts as a query against the manually annotated and non‐redundant SwissProt database (ftp://ftp.uniprot.org/pub/databases/uniprot/current_release/knowledgebase/complete/uniprot_sprot.dat.gz; release 2019_01, accessed 08/02/2019). A protein domain search was performed using “*hmmscan*” in HMMER v.3.1b2 against the Pfam‐A database v.31. Signal peptides were predicted using SignalP 4.1 (Petersen et al., 2011), transmembrane domains were annotated using TMHMM v.2.0 (Krogh et al., 2001), and rRNA transcripts were identified using RNAMMER v.1.2 (Lagesen et al., 2007).

For all remaining annotation strategies, we annotated the highest expressed isoform per gene. Protein families were identified using InterProScan v.5.36–75 (−appl Pfam ‐‐goterms) (Jones et al., 2014) and a BLASTP search (E‐value cutoff ≤1e‐3) against UniRef90 (ftp://ftp.uniprot.org/pub/databases/uniprot/uniref/uniref90/uniref90.fasta.gz; release version 31/07, accessed 08/08/2019) (UniProt Consortium, 2019). We further conducted local BLASTX searches (max_hsps 1, best_hit_overhang 0.1 and E‐value cutoff ≤1e‐5) against different protein databases. Two custom local protein BLAST databases were designed to specifically annotate putative detoxification genes of selected families: Cytochrome P450s (P450), carboxyl/cholinesterases (CCE), glutathione S‐transferases (GST), UDP‐glycosyltransferases (UGT), ATP‐binding cassette transporters (ABC transporters), and glucose‐methanol‐choline oxidoreductases (GMC). Additionally, arylsulfatase genes for which a specific member, glucosinolate sulfatase (GSS), evolved detoxifying properties in *P. xylostella*; and the NSP‐like gene family for which a member, nitrile‐specifier protein (NSP), evolved detoxifying properties within Pierinae (whites). The first protein database of these was compiled from detoxification genes of eight different reference Lepidoptera species as described in Breeschoten et al. (2019). This database was lacking representatives of GMCs, arylsulfatases (or GSS‐like genes) and NSP‐like genes and was only used to identify differentially expressed genes. The second database was an OrthoDB v.10 (Kriventseva et al., 2019) (accessed 08–11/2019) derived protein database using specific keywords (Table S5) including all selected detoxification gene families (overview detoxification genes in Table S6). A third database was constructed based on all Arthropoda protein sequences downloaded from the NCBI protein database (accessed, 31/01/2019). Annotation results can be found in Tables [Link], [Link], [Link], [Link], [Link], [Link], [Link], [Link], [Link], [Link], [Link], [Link], [Link], [Link], [Link], [Link], [Link], [Link], [Link], [Link], [Link], [Link], [Link], [Link], [Link], [Link], [Link], [Link], [Link], [Link], [Link], [Link], [Link].

### 2.7 Gene expression comparisons across four Noctuidae species

Putative orthologous genes were identified using OrthoFinder v.2.2.7 (Emms & Kelly, 2015) under default settings, using as input the amino acid sequences of the de novo assemblies according to the Transdecoder translations. Resulting orthogroups (OGs) were used in the species comparisons, forming a group of genes descended from a single gene in the last common ancestor for this group of species, and thus including both orthologs and paralogs. The OGs were annotated based on individual gene identifications (Table S13). OGs consisted of homologous genes, and in some cases the annotation strategy revealed different annotations for the genes clustered within a single OG (all annotations for each OG provided in Tables S13 and S21).

We applied the phylogenetic ANOVA method using the EVE model for studying gene expression evolution and population variance (Rohlfs & Nielsen, 2015). Phylogenetic ANOVA can detect genes with increased or decreased ratios of expression while taking phylogenetic relationships into account and controlling for a phylogenetic signal and therefore reduces bias caused by false positives. We used fold change (FC) values to compare expression levels of orthologous genes. Using FC instead of direct expression values circumvents cross‐species normalization (Dunn et al., 2013). We used the FC calculated by DESeq2 for pairwise sample comparisons between each plant treatment and the control group for each moth species individually. The FC values from orthologous genes of the different moths were then used for the between‐moth comparisons. We only included one‐to‐one orthologs (3369), also called strict orthologs (Fernández et al., 2020) without missing values for all samples. Clustering of the FC data matrix was done using “hclust” from the R package *stats* v.3.4.3 (R Development Core Team, 2020) to evaluate whether a phylogenetic signal was present in the expression data and, consequently, if the application of phylogenetic ANOVA using the EVE model was appropriate for our data.

Further, we employed Xspecies, a cross‐species meta‐analysis of gene expression (Kristiansson et al., 2013). Xspecies takes homology into account and compares expression data from genes with any number of orthologs and paralogs (Kristiansson et al., 2013). In short, this method compares the gene‐specific *p*‐values from the individual differential gene expression analysis within OGs between the moths.

For each moth species‐by‐plant treatment, the Xspecies program selects the lowest *p*‐value among all paralogs from each OG (i.e., as calculated from expression comparisons on the chosen plant vs. the control group). Each species is thus represented by one *p*‐value per OG. Based on these *p*‐values from the individual species, a single combined *p*‐value for each OG was calculated, which gives the level of significance for differential expression of homologous genes between the species. Because the method compares significance levels in terms of *p*‐values, it circumvents the need for normalization.

First, *p*‐values were extracted from the individual pairwise sample comparisons between each plant treatment and the control group as calculated with DESeq2. Thus, each moth species consisted of three samples, showing the significance of any expression differences: *Z. mays* vs. control, *B. oleracea* vs. control, and *N. tabacum* vs. control. In total, the dataset consisted of 9674 OGs with missing data allowed.

Based on the *p*‐values of homologous genes grouped in OGs, Xspecies calculates if the compared species show a difference in expression. The significance of this difference is calculated similar to Fisher's combined probability test and results in a *p*‐value describing the differential expression of homologous genes between the moth species. OGs were considered significantly differentially expressed between species with a false discovery rate (FDR) of ≤0.05. Heatmaps were created using ggplot2 in R (Wickham, 2016).

Finally, we have used Gene Ontology (GO) terms to compile annotated genes into broader functional terms. GO terms for all genes grouped in OGs were extracted from the species‐specific annotation report generated as part of the Trinotate pipeline. All GO terms of OGs associated with the selected species groups were compiled into broader terms, generic GO slim categories (subset used: goslim_generic.obo, the Gene Ontology Consortium; accessed August 2018), using the R package GOstats v.2.44.0 (Falcon & Gentleman, 2006).

## 3 RESULTS

### 3.1 Feeding assays

**FIGURE 1:**
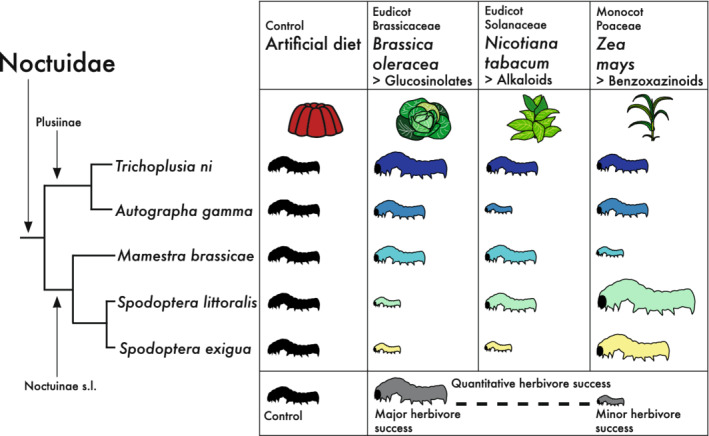
Results of the feeding assays comparing the growth rates (= herbivore success, a quantification for specialization and pest level) of larvae feeding on the control and three different host plant species: *Brassica oleracea*, *Nicotiana tabacum*, and *Zea mays* across the selected Noctuidae. In total, five moth species are included (*Spodoptera exigua*, *Spodoptera littoralis*, *Mamestra brassicae, Autographa gamma*, and *Trichoplusia ni*) for which the phylogenetic relatedness and higher taxonomic groups are shown on the left. Sizes represent herbivore success according to growth rate except for the control groups that are kept equal. Larval sizes reflect significant herbivore success differences between moth species. Size differences do not reflect significance for *S. exigua* and *A. gamma* feeding on *Z. mays* (*p*‐value = 5.2e‐2). As a consequence, sizes represent growth rate differences within moths except for *A. gamma* feeding on *B. oleracea* and *Z. mays* (*p*‐value = 4.8e‐2) and *M. brassicae* feeding on *N. tabacum* and *Z. mays* (*p*‐value = 4.9e‐1). For feeding assays results, see Tables S14 and S15).

Through our calculated growth rates (weight (mg)/developmental time (day)), we found that the selected Noctuidae larvae varied in their herbivore success across the chosen host plants (Figure 1; Tables S14 and S15). For *A. gamma*, the growth rates across all dietary treatments were significantly different, with larvae feeding on *Z. mays* showing the highest herbivore success (*N* = 73; growth rate: 0.235 ± 0.1 [mean ± SD] mg/day) (Table S15). *Mamestra brassicae* had the highest herbivore success on *B. oleracea* (*N* = 105; growth rate: 0.241 ± 0.12 mg/day) but was not significantly different from herbivore success on *N. tabacum* (*N* = 105; growth rate: 0.221 ± 0.18 mg/day) and the latter not from herbivore success on *Z. mays* (*N* = 98; growth rate: 0.174 ± 0.13 mg/day). Larvae of *T. ni* similarly were most successful on *B. oleracea* (*N* = 100; growth rate: 0.245 ± 0.1 mg/day), while exhibiting equivalent performance on *N. tabacum* and *Z. mays*. Finally, resembling results for the congener *S. exigua* (Breeschoten et al., 2019), *S. littoralis* exhibited highest herbivore success on *Z. mays* (*N* = 100; growth rate: 0.743 ± 0.41 mg/day). Intriguingly, performance on *Z. mays* diet, for *S. littoralis* larvae, was equivalent to performance on the control diet—a trend differing from that seen for the other moth species, whose performance was universally highest on the control diet.

To compare herbivore success between Noctuidae species (Figure 1), we normalized the species‐specific larval growth rates across moth species by comparing performance on each host plant to performance on the control diet. For the host plant *B. oleracea* herbivore success between *S. exigua*—*S. littoralis* and between *A. gamma*—*M. brassicae* showed no significant differences (Table S15). For *Z. mays*, there was no significant difference in herbivore success between *A. gamma*—*S. exigua* and between *A. gamma*—*T. ni*. Herbivore success difference between the Noctuidae species was much smaller for larvae feeding on *N. tabacum* than for the other host plants, and no significance was found for the species pairs: *A. gamma—S. exigua*, *S. littoralis—M. brassicae*, *S. littoralis—T. ni* and *M. brassicae—T. ni* (Table S15).

We were mainly interested in identifying genes potentially related to herbivore success. To enable this focus, we first—for each plant species—assigned moths into high vs. low herbivore success (i.e., larval performance) groups, avoiding single‐species comparisons, and studied these further by analyzing and comparing gene expression patterns for each plant treatment. For *B. oleracea*, the moths *T. ni*, *A. gamma*, and *M. brassicae* showed highest herbivore success in comparison with both *Spodoptera* species (Figure 1). For *N. tabacum*, the species *T. ni*, *M. brassicae*, and *S. littoralis* had the highest herbivore success. Finally, for *Z. mays* four different levels of herbivore success were found, and thus, we had to select three groups to avoid single‐species comparisons. We formed three groups of species with highest herbivore success: The first group consisted of *S. exigua* and *S. littoralis*, the second of *S. exigua, S. littoralis*, and *T. ni*, and finally, the third group consisted of *S. exigua, S. littoralis*, and *A. gamma*.

### 3.2 Expression quantification

DESeq2 was used for differential gene expression analysis. The differential gene expression analyses of *A. gamma* identified 14 clusters using a 50% cutoff of in total 1541 differentially expressed (DE) genes, for *M. brassicae* 12 clusters of 1429 DE genes, for *T. ni* 11 clusters of 1735 DE genes, and for *S. littoralis* 10 clusters of 4384 DE genes. The CPM, TMM cross‐sample normalized, and filtered count matrices are available in Tables [Link], [Link], [Link], [Link].

**FIGURE 2:**
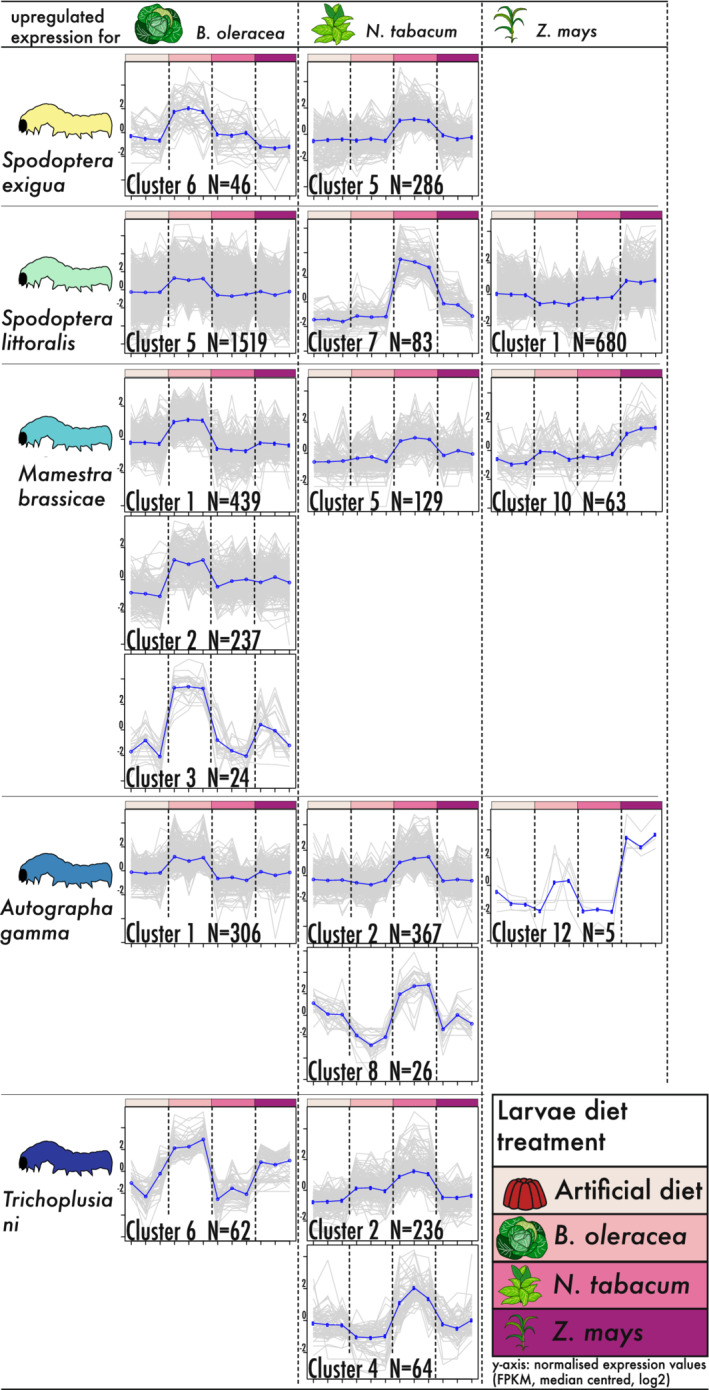
Selected diet treatment‐specific gene expression cluster plots. Shown are identified clusters with upregulated genes according to the differential gene expression analyses done for each species separately. Expression patterns were visualized and the number of genes (*N* = #) per cluster is indicated, with all samples represented on the x‐axis and the normalized expression values (FPKM, median centered, log2 transformed) on the y‐axis. All gene expression clusters are given in Figure [Link], [Link], [Link], [Link].

In our description of the DE genes, we focused on upregulated expression patterns because genes induced by plant feeding are potentially of importance to herbivory and insect pest formations. For *S. littoralis*, *A. gamma*, and *M. brassicae*, we identified clusters with upregulated genes for all three host plants. In contrast, for *T. ni* and *S. exigua* no cluster with upregulated genes was identified for *Z. mays* (Figure 2; Figures [Link], [Link], [Link], [Link]).

### 3.3 Multi‐species gene expression comparison

Orthologous genes were identified and grouped using OrthoFinder. The orthogroups (OGs) include genes descended from a single gene in the last common ancestor and therefore include orthologs and paralogs. OrthoFinder returned 52,921 OGs in total, with the largest OG including 99 genes and the smallest consisting of one single gene (Table S18).

**FIGURE 3:**
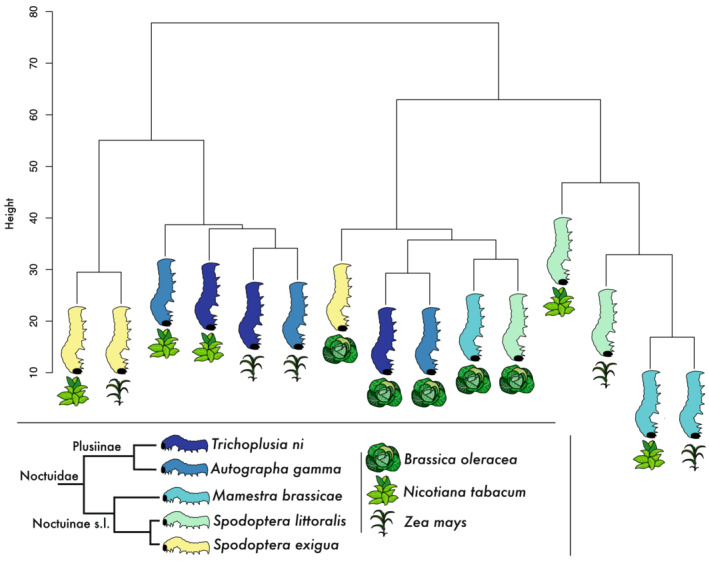
Hierarchical cluster plot of the fold change expression matrix including all identified strict orthologs (*N* = 3369) without missing data

The phylogenetic ANOVA method using the EVE model resulted in high *beta* values, which could indicate an absence of a phylogenetic signal (Table S19). The *beta* parameter gives the ratio of within‐species variance (diversity) to expression divergence between species. Given the high *beta* values, we used hierarchical clustering of the FC expression matrix in order to test the presence of a phylogenetic signal. Indeed, the hierarchical clustering approach did show an absence of a phylogenetic signal with species clustering more closely together according to host plant diet than their phylogenetic relatedness (Figure 3). Most notably, all moths feeding on *B. oleracea* clustered together on our gene expression dendrogram. In contrast to this, we did observe a potential phylogenetic pattern in which Plusiinae moth species (*T. ni* and *A. gamma*) clustered together on the gene expression dendrogram. This grouping—according to phylogenetic relatedness—could be indeed a result of phylogenetic signal inherent in the data. Nevertheless, the phylogenetic signal was too weak to be picked up by the statistical analysis (shared *beta* = 99.99), and thus, a phylogenetic model is not appropriate for our data. We, therefore, implemented Xspecies, a cross‐species gene expression analyses based on comparison of significance levels (Kristiansson et al., 2013). Xspecies identified in total 9675 significant OGs (Benjamini‐Hochberg false discovery rate (FDR) ≤0.05), of which 7728 OGs retrieved at least one annotation (Table S20).

**FIGURE 4:**
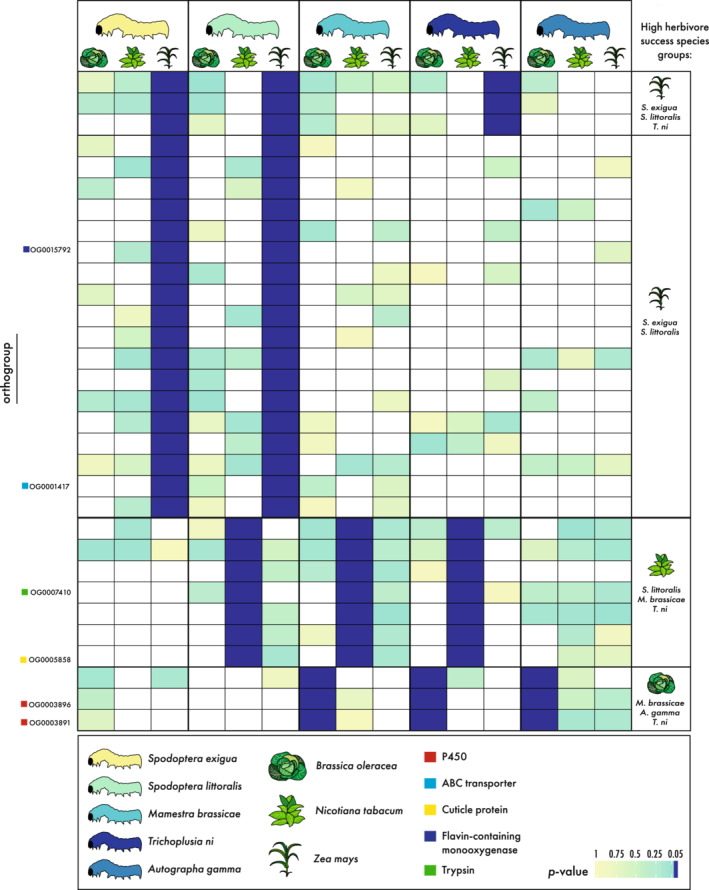
Selection for genes with expression patterns linked to high herbivore success (herbivore success species groups). Heatmap showing significantly differentially expressed homologous genes (grouped in orthogroups) between the moths as calculated with Xspecies. Genes are selected that show expression patterns related to high herbivore success for each host plant species (right). Genes are significantly upregulated in larvae feeding on the host plant as compared to the artificial food for those moths showing high herbivore success (≤0.05, dark blue). For moths with lowered herbivore success, these homologous genes do not show significant upregulation in larvae feeding on the host compared with the artificial food (yellow to green). Heatmap coloring corresponds to intensity of this difference by significance level of the *p*‐value (white corresponds to no expression). Various orthogroups are highlighted as discussed in the text. For a full list of annotated orthogroups, see Table S22.

With an interest in identifying genes underlying high herbivore success on the examined host plants, we focused on genes (i.e., OGs) with significant expression differences (relative to control diet) that were shared among successful herbivores on a given plant species (Figure 4).

For *Z. mays*, three different groups were selected—instead of a high/low‐performance distinction—due to avoidance of a single‐species comparison. Significant OGs were found for only two groups (Figure 4). The first species group, consisting of *S. littoralis*, *S. exigua*, and *T. ni*, had three OGs that were differentially expressed. A similar group, consisting of *S. littoralis*, *S. exigua*, and *A. gamma*, was lacking any significant OGs. The third group was a monophyletic assemblage of the two studied *Spodoptera* species (*S. exigua* and *S. littoralis*), which exhibited the highest ranked performances on *Z. mays*. This *Spodoptera* group had 18 OGs that were significantly differentially expressed. The most common generic GO slim categories associated with the third group included: biosynthetic process (GO:0009058), signal transduction (GO:0007165), cellular nitrogen compound metabolic process (GO:0034641), anatomical structure development (GO:0048856), response to stress (GO:0006950) and cellular protein modification process (GO:0006464) (Table S21).

The species group showing highest herbivore success on *N. tabacum* consisted of *S. littoralis, M. brassicae*, and *T. ni*. Among the species in this high herbivore success group, a total of seven OGs showed uniquely shared expression shifts. For the high‐performing species on *B. oleracea* (*M. brassicae*, *T. ni*, and *A. gamma*), only three OGs showed unique significance (i.e., significant expression shifts in these three moth species on *B. oleracea*, relative to controls, but in neither of the two remaining moth species) (Figure 4). The number of GO terms associated with these species groups was too few to be compiled into broader generic GO slim categories. However, the majority of the genes in all OGs are involved in general physiological, developmental, and metabolic functions while various OGs belong to gene families involved in digestion and detoxification (Table S22).

Focusing on detoxification genes within these high herbivore success species groups, only three OGs were annotated as putative detoxification genes. First, on *Z. mays*, one ABC OG was uniquely, differentially expressed among the successful herbivore group. Further, two P450 genes were uniquely differentially expressed for the successful herbivore group on *B. oleracea* (Table S22).

**FIGURE 5:**
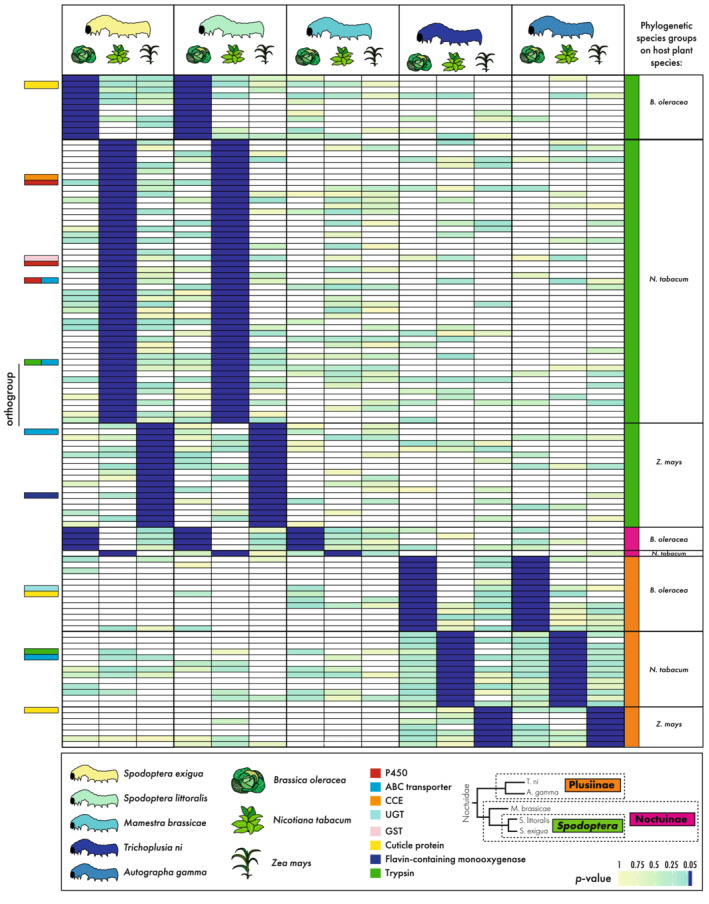
Selection for genes with expression patterns potentially caused by evolutionary history (phylogenetic species groups). Heatmap showing significantly differentially expressed homologous genes (grouped in orthogroups) between the moths as calculated with Xspecies. Genes are selected that show expression patterns that can be linked to evolutionary history for each host plant species independently (right). The tested phylogenetic species groups are depicted in the phylogeny (lower right) with colors coding for each clade that link to the species groups in the heatmap (green = *Spodoptera*, pink = Noctuinae, and orange = Plusiinae). Genes are significantly upregulated in larvae feeding on the host plant as compared to the artificial food for those moths within the phylogenetic species groups (≤0.05, dark blue), while for larvae outside the clades these homologous genes are not significantly different expressed between the host plant and artificial food (yellow to green). Heatmap coloring corresponds to intensity of this difference by significance level of the *p*‐value (white corresponds to no expression). Various orthogroups are highlighted as discussed in the text. For a full list of annotated orthogroups, see Table S22.

Besides high herbivore success, we grouped the significantly differentially expressed genes according to phylogenetic relatedness (Figure 5). This revealed expression patterns, which are likely caused by shared evolutionary histories and could therefore not be correlated with herbivore success alone. We selected three phylogenetic species groups, *Spodoptera* (*S. exigua* and *S. littoralis*), Noctuinae (*S. exigua, S. littoralis*, and *M. brassicae*) and Plusiinae (*T. ni* and *A. gamma*). For each phylogenetic species group, we selected significantly differentially expressed OGs per host plant species, again based on the Xspecies results. The homologous genes selected within each phylogenetic clade proved significantly differentially expressed in moths feeding on the host plant compared with the control food, while insignificant (*p* > 0.05) for the remaining moth species outside the clade. This is similar to the high herbivore success selection criterion and does reveal expression patterns related to specific host plant species response, in this case shared among related species.

For the phylogenetic species group *Spodoptera*, 18 OGs were significantly differentially expressed in their response to feeding on *Z. mays* as compared to the control food. Most common generic GO slim categories associated with these 18 OGs included anatomical structure development (GO:0048856), cell cycle (GO:0007049), biosynthetic process (GO:0009058), signal transduction (GO:0007165), and cellular nitrogen compound metabolic process (GO:0034641) (Table S21). Further, for the *Spodoptera* species group 11 OGs were significantly differentially expressed when feeding on *B. oleracea*. Two GO slims were most commonly associated with these OGs: biosynthetic process (GO:0009058) and cellular nitrogen compound metabolic process (GO:0034641) (Table S21). Finally, 49 OGs were significantly differentially expressed when feeding on *N. tabacum*, which were associated with three GO slim categories: cellular nitrogen compound metabolic process (GO:0034641), response to stress (GO:0006950), and DNA metabolic process (GO:0006259) (Table S21). The species within the phylogenetic group Noctuinae had no OGs significantly differentially expressed when feeding on *Z. mays*, while four OGs were significantly differentially expressed when feeding on *B. oleracea* and one single OG significantly differentially expressed when feeding on *N. tabacum*. Only for the OG unique to high performance on *N. tabacum*, five associated GO slim categories were identified, including biosynthetic process (GO:0009058), carbohydrate metabolic process (GO:0005975), cellular protein modification process (GO:0006464), lipid metabolic process (GO:0006629), and cellular nitrogen compound metabolic process (GO:0034641) (Table S21). Finally, for the Plusiinae species group seven OGs were significantly differentially expressed when feeding on *Z. mays*. The major associated GO slim categories were biosynthetic process (GO:0009058), cellular nitrogen compound metabolic process (GO:0034641), and immune system process (GO:0002376) (Table S21). A similar total of 13 OGs were significantly differentially expressed when feeding on *B. oleracea* or on *N. tabacum*. The major associated GO slim categories for Plusiinae feeding on *B. oleracea* included biosynthetic process (GO:0009058), small molecule metabolic process (GO:0044281) and lipid metabolic process (GO:0006629), and on *N. tabacum* included cellular protein modification process (GO:0006464), signal transduction (GO:0007165) and anatomical structure development (GO:0048856) (Table S21).

Multiple OGs in these phylogenetic species groups are identified as detoxification gene family members. Within the *Spodoptera* group feeding on *N. tabacum* one OG is identified as a CCE family member, three OGs as putative P450s, one GST, and one ABC transporter. Similar to the high herbivore success species group, one OG was identified as ABC in *Spodoptera* feeding on *Z. mays*. Only a single OG annotated as UGT is differentially expressed within Plusiinae feeding on *B. oleracea* compared with the other species and one ABC transporter within Plusiinae on *N. tabacum* (Table S22).

Except the high herbivore success group on *Zea mays* consisting of both closely related *Spodoptera* species, there are no OGs shared between high herbivore success species groups and phylogenetic species groups. The high herbivore success group on *Zea mays* (consisting of *S. exigua* and *S. littoralis*) is identical to the phylogenetic species group *“Spodoptera*” feeding on *Z. mays*. Distinguishing between herbivory success and evolutionary history (phylogeny) is not possible in this case. The expression of these genes may play important roles in the feeding success of *Spodoptera* on *Z. mays* and/or may be due to shared evolutionary history.

## 4 DISCUSSION

We performed a multi‐species comparison of gene expression patterns of larvae of five different cutworm moth species (Noctuidae), feeding, and developing on three different host plant species. The polyphagous Noctuidae had different herbivore success rates per host plant species as quantified by feeding assays (Figure 1; Tables S14 and S15). In general, polyphagous herbivore insect species showed a level of host plant species specialization (Schoonhoven et al., 2005), which eventually could lead to pest formations. In order to understand this specialization, we focused on transcriptional plasticity related to high herbivore success.

To study and interpret gene expression data in a biological and evolutionary context, comparable data from closely related species need to be collected and analyzed within a multi‐species comparison implementing the phylogenetic perspective (Dunn et al., 2013). Failing to incorporate phylogenetic relationships in a multi‐species comparison can result in misleading and/or erroneous conclusions (Dunn et al., 2018). The explicit phylogenetic framework applied here discriminated between gene expression patterns shared as a result of evolutionary history from those expression patterns independent from their phylogenetic relationships. Consequently, our applied multi‐species comparison revealed shared and unique gene expression patterns of the five different moth species while feeding on different host plants regardless of their evolutionary history.

### 4.1 Feeding assays

Herbivore success, measured as larval performance, for the different host plants differed among the five species (Figure 1), although all five moth species are polyphagous herbivores able to feed on the selected plants (EPPO, 2019). Herbivore success of polyphagous insect species often varies within the range of host plant species they feed on (Schoonhoven et al., 2005). This is also evident from our feeding assay results: The levels of herbivore success for the selected host plant species are variable and species‐specific. Therefore, we have focused on the transcriptional response behind this variation in herbivore success. The response to host plants can be influenced by host plant experience of parent generations (Müller et al., 2017). To reduce this bias, we have excluded insect parent host plant experience as a cause for the difference by first rearing the paternal generation of all moth species on the control diet (e.g., no anti‐herbivory compounds). Nevertheless, the stock rearing of the species in the comparisons did vary. However, our interest lies in the difference in response to host plant feeding. The history of any species or population in comparative studies will vary, which is tested by implementation of feeding assays. All further rearing and feeding experiments were standardized.

### 4.2 Expression data analysis and transcript annotation

For the individual moth DE analyses, we focused on upregulated genes due to their importance in coping with host plants for which larvae have low levels of herbivore success (Breeschoten et al., 2019). Differential gene expression analyses within the individual moth species revealed diet‐specific gene clusters for all host plants and the artificial food in *S. littoralis, A. gamma*, and *M. brassicae* (Figure 2). These clusters of upregulated genes indicate a flexible transcriptional response influenced by diet. Plant‐specific transcriptional responses have been observed in several polyphagous insects, including Lepidoptera (Breeschoten et al., 2019; Celorio‐Mancera et al., 2012), Coleoptera (Müller et al., 2017), Hemiptera (Mathers et al., 2017), and other arthropods such as the spider mite *Tetranychus urticae* (Grbić et al., 2011).

In an earlier study, we found that *S. exigua* relies on non‐diet‐specific expressed genes when feeding on host plants with optimal suitability, showing high herbivore success (Breeschoten et al., 2019). Larvae of *S. exigua* feeding on *Z. mays* showed high herbivore success and absence of diet‐specific gene clusters, indicating that detoxification and digestion was potentially sufficient with a general gene activity. This “specialization” and absence of a diet‐specific cluster shares similarities with the transcriptional responses of monophagous species to their host plants. Monophagous species show a host plant‐specific transcriptional response, often with evolved adaptations, to efficiently detoxify the defenses of their hosts (Heidel‐Fischer et al., 2019; Wheat et al., 2007). Moreover, molecular studies have shown that monophagous species generally employ a lower number of expressed genes compared with polyphagous species feeding on host plant species with similar defenses (Govind et al., 2010; Ragland et al., 2015; Roy et al., 2016; Schweizer et al., 2017), relying more on a constitutive and targeted response (Berenbaum, 2002).

The ability of a polyphagous species to feed on various plants is potentially due to a greater transcriptional plasticity than of a monophagous insect (Birnbaum & Abbot, 2020). For example, the polyphagous spider mite (*T. urticae*) showed a threefold increase in DE genes when shifting to a new and thus less adapted/optimal host plant (Dermauw, Wybouw, et al., 2013). Thus, the absence of a diet‐specific expression cluster in *S. exigua* feeding on *Z. mays* might be due to “specialization” for this host plant (Breeschoten et al., 2019). However, this non‐diet‐specific response seems to be species‐specific to *S. exigua*. For example, *S. littoralis* showed also the highest herbivore success on *Z. mays* (Figure 1), but in contrast to *S. exigua* we could identify for *S. littoralis* a large number of upregulated genes (Figure 2). In fact, *S. littoralis* showed the largest number of upregulated genes in combination with highest herbivore success on *Z. mays*. We conclude that the number of induced genes, as an adaptive response to an optimal host plant, is rather species‐specific and there is no general pattern within polyphagous Noctuidae. However, the species‐specific transcriptional plasticity might enable the wide host plant usage of polyphagous insects (Birnbaum & Abbot, 2020).

The expression of detoxification genes within diet‐dependent gene clusters is of particular interest in insect–plant interaction studies (further discussed in File S23). For each moth species, we focused on the expression of eight gene families (P450, CCE, GST, UGT, ABC, GMC, NSP‐like gene family and GSS‐like genes) involved in detoxification of plant defense compounds (Heidel‐Fischer & Vogel, 2015; Kant et al., 2015; Li et al., 2007). The detoxifying properties of these gene families (except NSP and GSS) are not restricted to specific species but generally recognized in herbivorous insects (Heckel, 2018; Kant et al., 2015; Zhang et al., 2013). Detoxification activity of nitrile‐specifier protein (NSP) and glucosinolate sulfatases (GSS) is known from specific lepidopteran taxa outside the Noctuidae, namely the genus *Plutella* and subfamily Pierinae. In Pierinae (whites), specialized on glucosinolate‐containing crucifers, a NSP gene evolved to modify the toxic degradation products of glucosinolates into less toxic nitriles (Fischer et al., 2008; Wheat et al., 2007; Wittstock et al., 2004). The NSP‐like gene family is an insect‐specific family, but only in Pierinae members an NSP gene evolved a glucosinolate detoxifying mechanism (Fischer et al., 2008; Wheat et al., 2007). For the NSP‐like family, we identified gene members in all five Noctuidae species, but none was differentially expressed (see NSP, Table S6). As expected (Fischer et al., 2008; Wheat et al., 2007), these results suggest the absence of NSP‐mediated detoxification of *B. oleracea‐*derived defensive compounds among the studied Noctuidae moths.

In a separate family (Plutellidae), the diamondback moth (*Plutella xylostella*) evolved a GSS enzyme, which is part of an arylsulfatase class of genes that prevents the formation of toxic hydrolysis products by Brassicaceae plants (Heidel‐Fischer et al., 2019; Ratzka et al., 2002). We looked at the expression of arylsulfatase genes, but as reported for NSP, we found a host‐specific response was absent in all Noctuidae even though members were differentially expressed in *T. ni* and *S. littoralis* (see GSS, Table S6).

Glucose‐methanol‐choline oxidoreductases (GMCs) were identified in all moth species feeding on multiple host plants (see GMC, Table S6). Oxidoreductases have been described to degrade various groups of plant specialized metabolites (Müller et al., 2017; Zhang et al., 2013). According to our results, there is no evidence for a host plant species‐specific expression of GMCs or herbivore success‐related response. The GMCs seem to be involved in a general degradation of specialized metabolites of plants. However, oxidoreductases have been associated with immunity (Sun et al., 2012), which would also explain the observed wide expression pattern.

In examining the remaining gene classes with potential detoxifying function, we found that all studied Noctuidae upregulated various genes from the P450, CCE, GST, UGT, and ABC functional classes when feeding on *B. oleracea*, with just two exceptions. *Spodoptera exigua*, lacking significantly upregulated P450s, and *T. ni*, lacking upregulated CCEs (Table S6). Since both P450s and CCEs are involved in hydrolysis, detoxification of glucosinolates could potentially be dominated by a single family. The main defense strategy of *B. oleracea* against herbivores is by the formation of toxins like isothiocyanates (ITCs) after activation of glucosinolates by myrosinases (e.g., due to chewing) (Kliebenstein et al., 2005). Many different forms of counteradaptations by herbivore insects have been described (Jeschke et al., 2016, 2017). The general detoxification strategy, besides specialized responses like sequestration (Beran et al., 2014; Zagrobelny & Møller, 2011), involves diverse gene families from the detoxification pathway (Jeschke et al., 2016). Detoxification can potentially take place by direct conjugation (phase II) of ITC due to its highly reactive state and skipping the first phase of activating the metabolite (Jeschke et al., 2016; correspondence Katharina Schramm, November 2020). This could explain the absence of upregulated P450s or CCEs in *S. exigua* and *T. ni* in moths feeding on *B. oleracea*. Gene members of the GST family were found upregulated in all moths feeding on *B. oleracea* (Table S6), which supports the suspected role of GSTs in the conjugation reaction (Jeschke et al., 2016; Schramm et al., 2012). However, several studies showed that a different detoxification strategy by polyphagous moths is potentially through direct conjugation of ITC by L‐glutathione (GSH) (Jeschke et al., 2016; Schramm et al., 2012). It was shown that this conjugation mechanism is possibly shared among many polyphagous Noctuidae species, including *M. brassicae*, *T. ni, S. exigua*, and *S. littoralis* based on glutathione conjugates in larval feces (Jeschke et al., 2017; Schramm et al., 2012). Also, the clustering of all Noctuidae species in the *B. oleracea* diet treatment using the fold change (FC) expression matrix (Figure 3) further indicates a shared response to the toxins employed by *B. oleracea* and might indicate a shared ITC conjugation process.

#### 4.2.1 Expression comparison using Xspecies

The main aim of our study was to evaluate whether successful herbivory is determined by a shared or lineage‐specific transcriptional response. To address this question, we have used a comparative phylogenetic framework and compared the expression patterns using a cross‐species gene expression analyses based on comparison of significance levels (Kristiansson et al., 2013), including up‐ and downregulated homologous genes.

From the significantly differentially expressed homologous genes (grouped in OGs) as calculated with Xspecies, we selected for each host plant OGs with shared expression patterns related to high herbivore success (see results for selection details) (Figure 4).

The OGs identified showed that moths feeding with high herbivore success on host plants employ host plant‐specific OGs (Figure 4; Table S22). We further focused on the shared expression of detoxification genes for species with high herbivore success (=high herbivore success species groups), given the important role of detoxification in successful plant feeding and resisting specialized metabolites.

Three OGs within the high herbivore success species groups belong to one of the analyzed detoxification gene families (P450, CCE, GST, UGT, and ABC) (Figure 4). This low number of “detoxification OGs” with shared response indicates that expression of detoxification gene family members was highly species‐specific because each moth did transcribe a larger number of DE detoxification genes (Table S6). Nevertheless, the shared response of the three OGs could be correlated with host plant adaptation of species with high herbivore success. Two OGs were annotated as P450 members, a gene family broadly involved in detoxification (Feyereisen, 2005; Schuler, 2011). Activity of these genes could be associated with high herbivore success of *M. brassicae, A. gamma*, and *T. ni* while feeding on *B. oleracea* (Figure 4). Further, we found one uniquely expressed OG, in successful herbivores, to encode ABC transporters, which belong to a gene family involved in the detoxification pathway (Bretschneider et al., 2016; Dermauw, Osborne, et al., 2013; Dermauw & Van Leeuwen, 2014). This OG was significantly upregulated in both *Spodoptera* species while feeding on *Z. mays* with high herbivore success. However, given the shared evolutionary history of these species, we could not correlate the activity of this OG to herbivore success alone (Figures 4 and 5).

Other gene families, aside from the commonly studied detoxifying families discussed above, could also play key roles in high herbivore success. For example, OG0015792, annotated as Flavin‐containing monooxygenase (FMO) based on Pfam domains and senecionine N‐oxygenase based on the Uniref90 annotation (Table S22), was significantly differentially expressed in both, *S. exigua* and *S. littoralis* while feeding on *Z. mays* with high herbivore success. Its expression was minimal to absent in the other studied species, hinting at a lineage‐specific response to this particular host plant (Figure 4). The FMOs are known to oxidize xenobiotics in a similar way as P450s (Eswaramoorthy et al., 2006) and are involved in alkaloid detoxification (Lindigkeit et al., 1997; Naumann et al., 2002). The activity of the FMO‐annotated gene members in OG0015792 might indicate a potential role in herbivory of Noctuidae.

We also selected host plant species‐specific expression patterns that are found only within three phylogenetic species groups (*Spodoptera*, Noctuinae, and Plusiinae). These expression patterns were likely caused by shared evolutionary history, and as a consequence might not be purely correlated with herbivore success (Figure 5). The number of shared OGs in phylogenetic species groups (average = 15 OGs) was larger than within high herbivore success species groups (average = 4 OGs), indicating the importance of lineage‐specificity for host plant response. However, the larger number of shared OGs was not found for the Noctuinae groups (*S. exigua, S. littoralis*, and *M. brassicae*; 4 OGs for *B. oleracea*, 1 OG for *N. tabacum*, and none for *Z. mays*; Figure 5), which could be explained by the larger phylogenetic distance due to inclusion of a third distant‐related species (*M. brassicae*).

Of the annotated detoxification gene families, all families (P450, CCE, UGT, GST, and ABC) were present in the shared OGs of the phylogenetic species groups (Figure 5). However, the detoxification gene families CCE, GST, and UGT were represented by a single OG only. Indeed, based on the individual moth differential expression analysis we identified many DE detoxification genes (Table S6). However, the shared response of only a single OG of these gene families within phylogenetic species groups (or three or four OGs for P450 and ABC) did indicate that the individual moths rely for a large degree on a species‐specific employment of detoxification genes.

Most gene families showing differential expression have no clear role in detoxification based on the annotations (Table S22). Indeed, various OGs were involved in processes like physiological, metabolic, and developmental functions, indicating a potential impact of host plant feeding on these processes. However, a shared transcriptional response of homologous genes from these gene families, both within high herbivore success species groups (Figure 4) or within phylogenetic species groups (Figure 5), could indicate a role in herbivory and potential pest formations. The role of gene families not known to be involved in detoxifying properties has shown to be potentially important in facilitating feeding on newly introduced host plant species (Dermauw, Wybouw, et al., 2013; Wybouw et al., 2015). Indeed, various gene families were showing joint differential expression within high herbivore success species groups and phylogenetic species groups.

An example of a widely occurring gene family is the trypsins, which are involved in the digestion of plant material by protein hydrolysis (Muhlia‐Almazán et al., 2008). The unique occurrence of the trypsin homologs containing OG (OG0007410), identified in the high herbivore success species group consisting of *S. littoralis*, *M. brassicae*, and *T. ni*, may indicate important digestive roles of trypsins while feeding on *N. tabacum* (Figure 4). However, trypsin family members were also widely identified within all individual moth gene expression analyses (Table S7–S12). Also within the two phylogenetic species groups, *Spodoptera* and Plusiinae, trypsin family members were shared while feeding on *N. tabacum* (OG0020891 and OG0008359; Figure 5). Indeed, the wide expression of trypsins within all moth species indicated a non‐host‐specific importance for herbivory.

We observed a similar pattern for the insect cuticle protein family. A strengthened cuticle of the peritrophic matrix and midgut, which comes into contact with toxins and abrasive food particles, may improve the protective function by forming a protective physical barrier for biochemical toxins (Agrawal et al., 2014; Hegedus et al., 2009; Kelkenberg et al., 2015; Kumar et al., 2018). Differential expression of transcripts coding for structural constituents of the cuticle in response to the diet is found in larvae of various herbivorous insect species (Breeschoten et al., 2019; Celorio‐Mancera et al., 2013; Hoang et al., 2015; Müller et al., 2017; Orsucci et al., 2018). In our multi‐species comparison, we identified three OGs as members of the insect cuticle protein family. However, only one OG can be associated with high herbivore success on *N. tabacum* (OG0005858, Figure 4). The two other OGs (OG0011643 and OG0007820, Figure 5) share lineage‐specific expression within the phylogenetic species group Plusiinae. In general, the wide occurrence of certain families, such as trypsin and cuticle protein families, shows their importance for herbivory. The employment seems highly lineage and species‐specific. Only, the expression of a few specific OGs of these gene families can be associated with high herbivore success. These genes are of high interest for future studies aiming for a deeper understanding of the genetic mechanisms of potential pest formations of Noctuidae moths.

## 5 CONCLUSIONS

We studied the gene expression of polyphagous Noctuidae species feeding on different host plants associated with varying levels of herbivore success as quantified with feeding assays. Our work shows how major polyphagous insects rely on the deployment of some widely employed gene families, indicating their importance for herbivory and polyphagy. However, transcriptional plasticity was high and moth species‐specific. By implementation of a phylogenetic framework, we identified groups of homologous genes with shared expression within clades of related species (*Spodoptera*, Plusiinae and Noctuinae). Furthermore, we identified shared expression patterns of homologous genes between moth species associated with high herbivore success, independent of phylogeny and thus indicating convergence.

Our main aim was to evaluate whether successful herbivory is determined by a shared or lineage‐specific transcriptional response. We conclude that successful polyphagous herbivores, or potential pests, have shared expression of groups of homologous genes but rely also on species‐specific transcriptional plastic expression. The shared expression indicated the potential role of these genes in reaching high herbivore success on specific plants. Clearly, similar comparative studies are needed to verify the shared gene activity in related (polyphagous) insect clades forming pests. As a whole, our results provide an initial overview of the genetic basis of polyphagy and pest formations. The lineage‐specific shared expression, putatively important in plant feeding among related species, is of interest for the understanding of the evolution and genetic basis of polyphagy. Pinpointing the shared expression of genes associated with high herbivore success is a promising step toward the development of sustainable ways of coping with, and genetic understanding of, herbivorous insects forming pests.

## AUTHOR CONTRIBUTIONS

**Thijmen Breeschoten:** Conceptualization (equal); data curation (lead); formal analysis (lead); investigation (equal); methodology (equal); software (lead); visualization (lead); writing – original draft (lead); writing – review and editing (equal). **M. Eric Schranz:** Conceptualization (equal); investigation (equal); methodology (equal); project administration (equal); supervision (equal); writing – review and editing (equal). **Erik Poelman:** Resources (supporting); writing – review and editing (supporting). **Sabrina Simon:** Conceptualization (equal); investigation (equal); project administration (equal); software (equal); supervision (equal); writing – original draft (equal); writing – review and editing (equal).

## Supporting information


Table S1

Table S2

Table S3

Figure S4a

Figure S4b

Figure S4c

Figure S4d

Table S5

Table S6

Table S7a

Table S7b

Table S7c

Table S7d

Table S8a

Table S8b

Table S8c

Table S8d

Table S8e

Table S9a

Table S9b

Table S9c

Table S9d

Table S9e

Table S10a

Table S10b

Table S10c

Table S10d

Table S10e

Table S10a2

Table S10b2

Table S10c2

Table S10d2

Table S10e2

Table S11a

Table S11b

Table S11c

Table S11d

Table S11e

Table S12a

Table S12b

Table S12c

Table S12d

Table S12e

Table S13

Table S14

Table S15

Table S16a

Table S16b

Table S16c

Table S16d

Figure S17a

Figure S17b

Figure S17c

Figure S17d

Table S18

Table S19

Table S20

Table S21

Table S22

File S23

## Data Availability

All Supplementary Data are uploaded to the 4TU Centre for Research Data repository and available online DOI:10.4121/14115386. All supplementary datafiles are uploaded, totaling 60 files, and include all measurements, results of the statistical analyses, raw and analyzed expression values, and annotation information. Further, all genomic material is available at the NCBI database under BioProject accession numbers: PRJNA512462 (*S. littoralis*), PRJNA556816 (*A. gamma*), PRJNA543970 (*M. brassicae*), and PRJNA548256 (*T. ni*) (https://www.ncbi.nlm.nih.gov/bioproject/). Genetic material is stored in −80°C storage facility of the Biosystematics Group, Wageningen University & Research.
