# Supplementary figures and images for: Family dinner: Transcriptional plasticity of five Noctuidae (Lepidoptera) feeding on three host plant species
Source: Ecol Evol. 2022 Sep 6;12(9):e9258. doi: 10.1002/ece3.9258 (PMC9448971; doi:10.1002/ece3.9258)

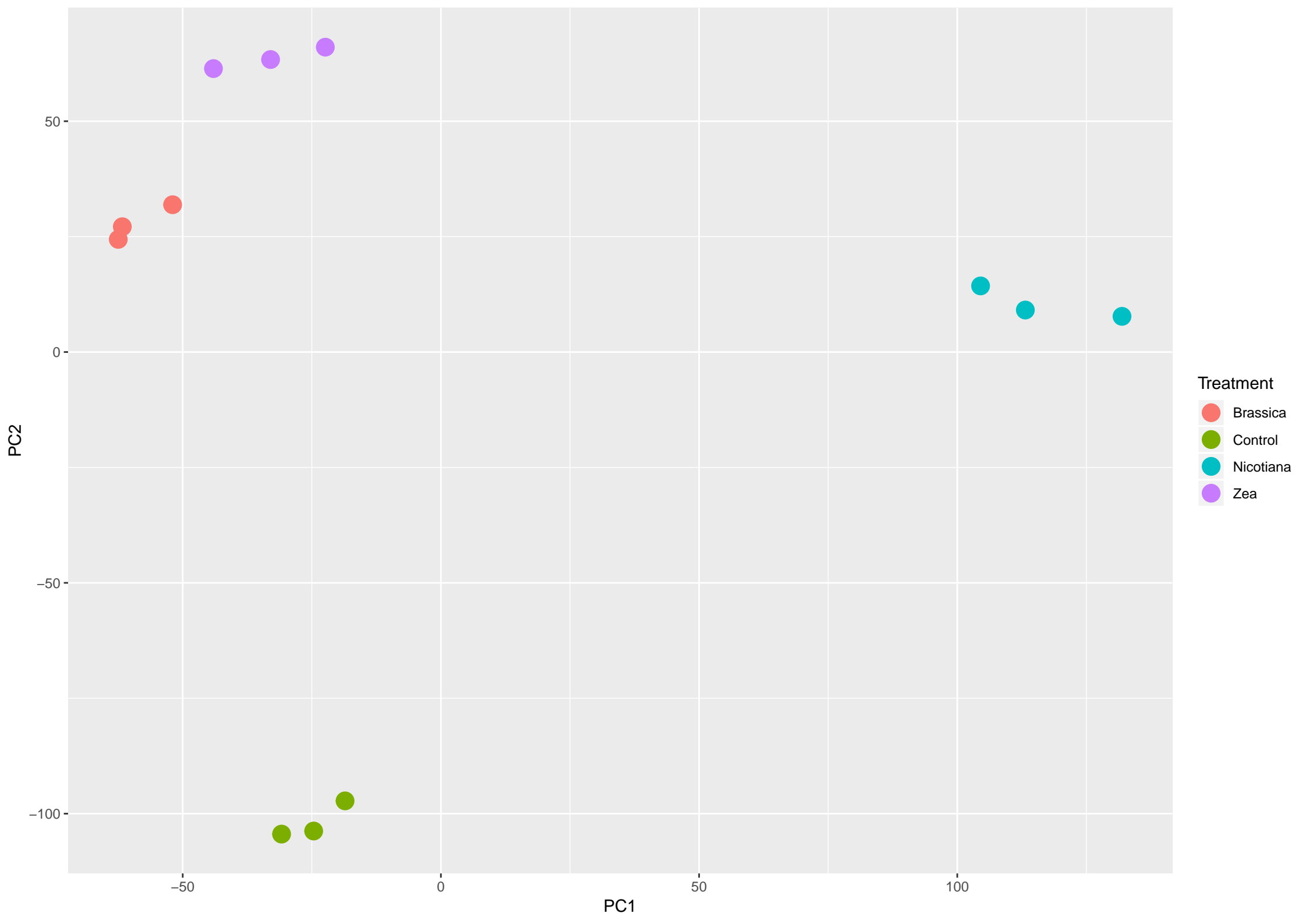

Supplement: Supplementary file 4 — Figure S4a [file ECE3-12-e9258-s040.pdf]

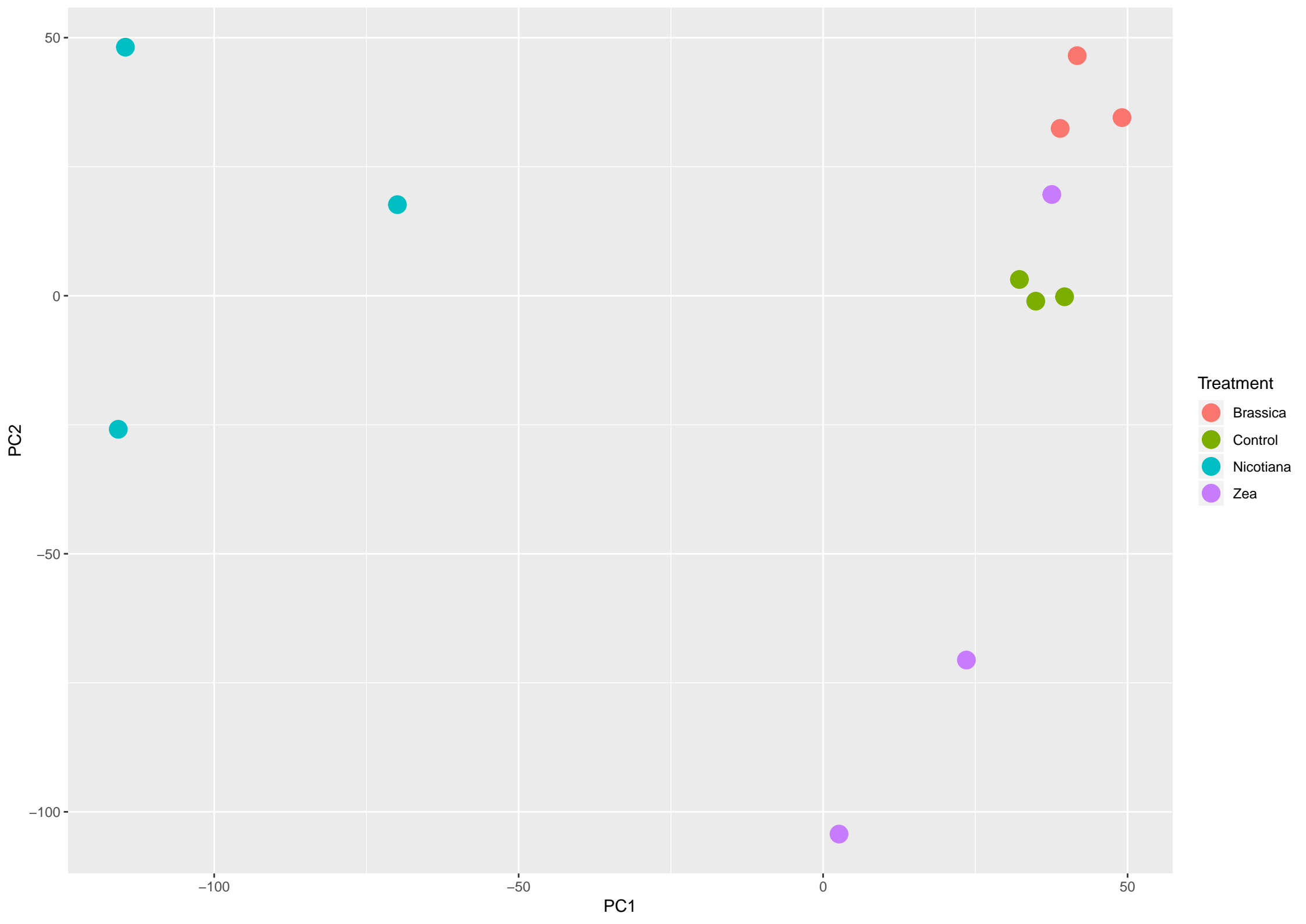

Supplement: Supplementary file 5 — Figure S4b [file ECE3-12-e9258-s015.pdf]

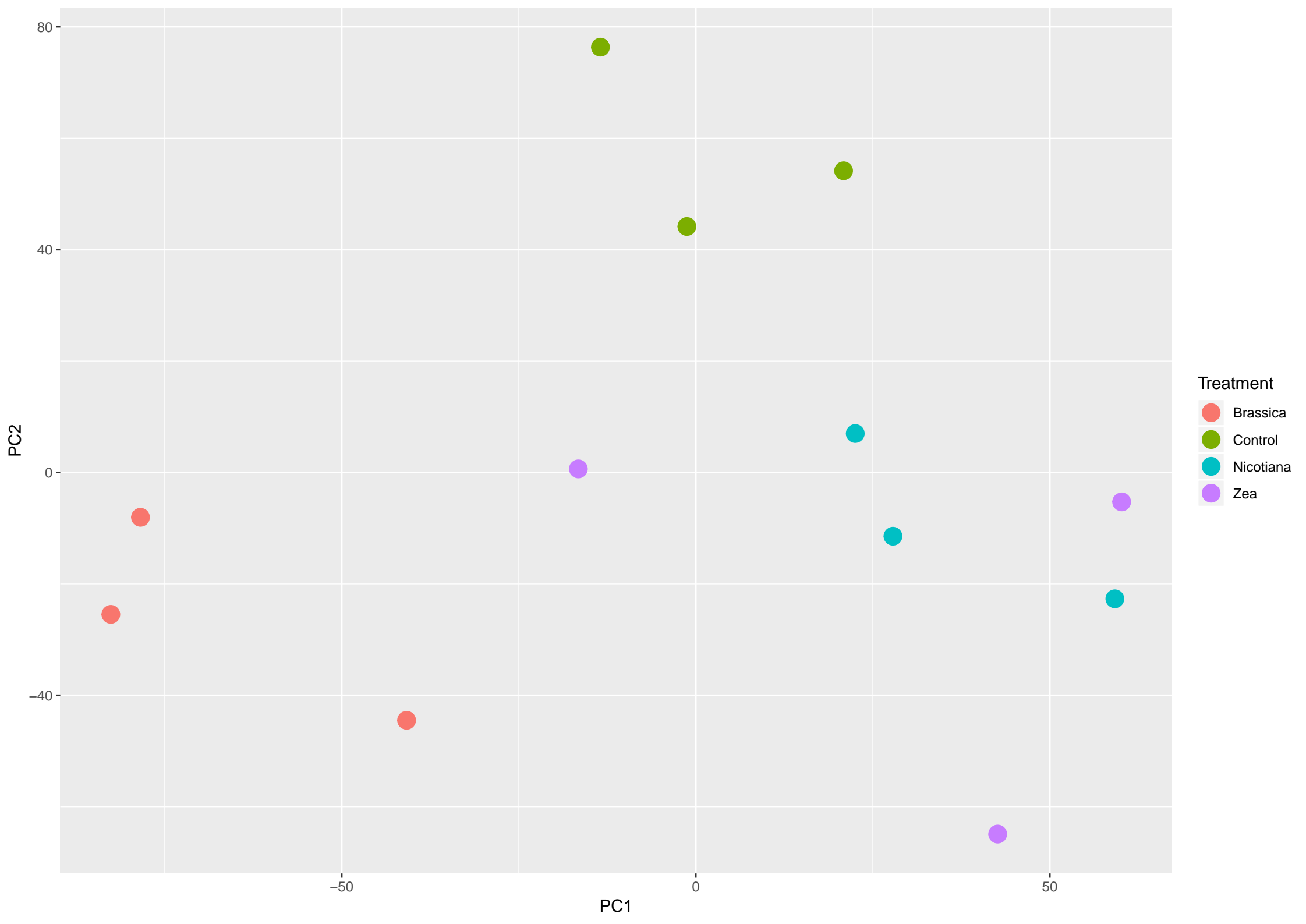

Supplement: Supplementary file 6 — Figure S4c [file ECE3-12-e9258-s043.pdf]

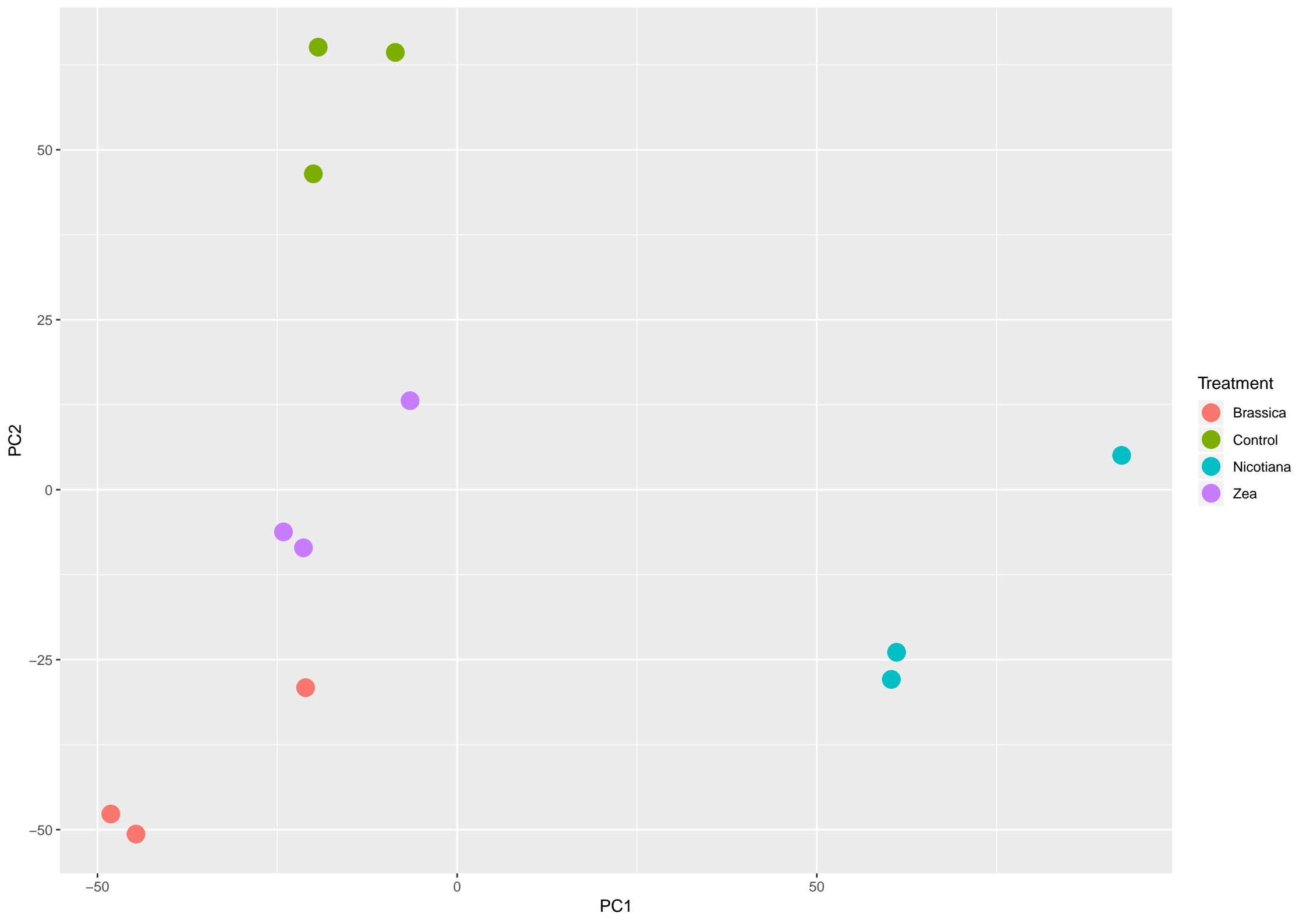

Supplement: Supplementary file 7 — Figure S4d [file ECE3-12-e9258-s049.pdf]
