# Supplementary material for: Family dinner: Transcriptional plasticity of five Noctuidae (Lepidoptera) feeding on three host plant species
Source: Ecol Evol. 2022 Sep 6;12(9):e9258. doi: 10.1002/ece3.9258 (PMC9448971; doi:10.1002/ece3.9258)
Supplement: Supplementary file 51 — Figure S17a [file ECE3-12-e9258-s051.pdf]

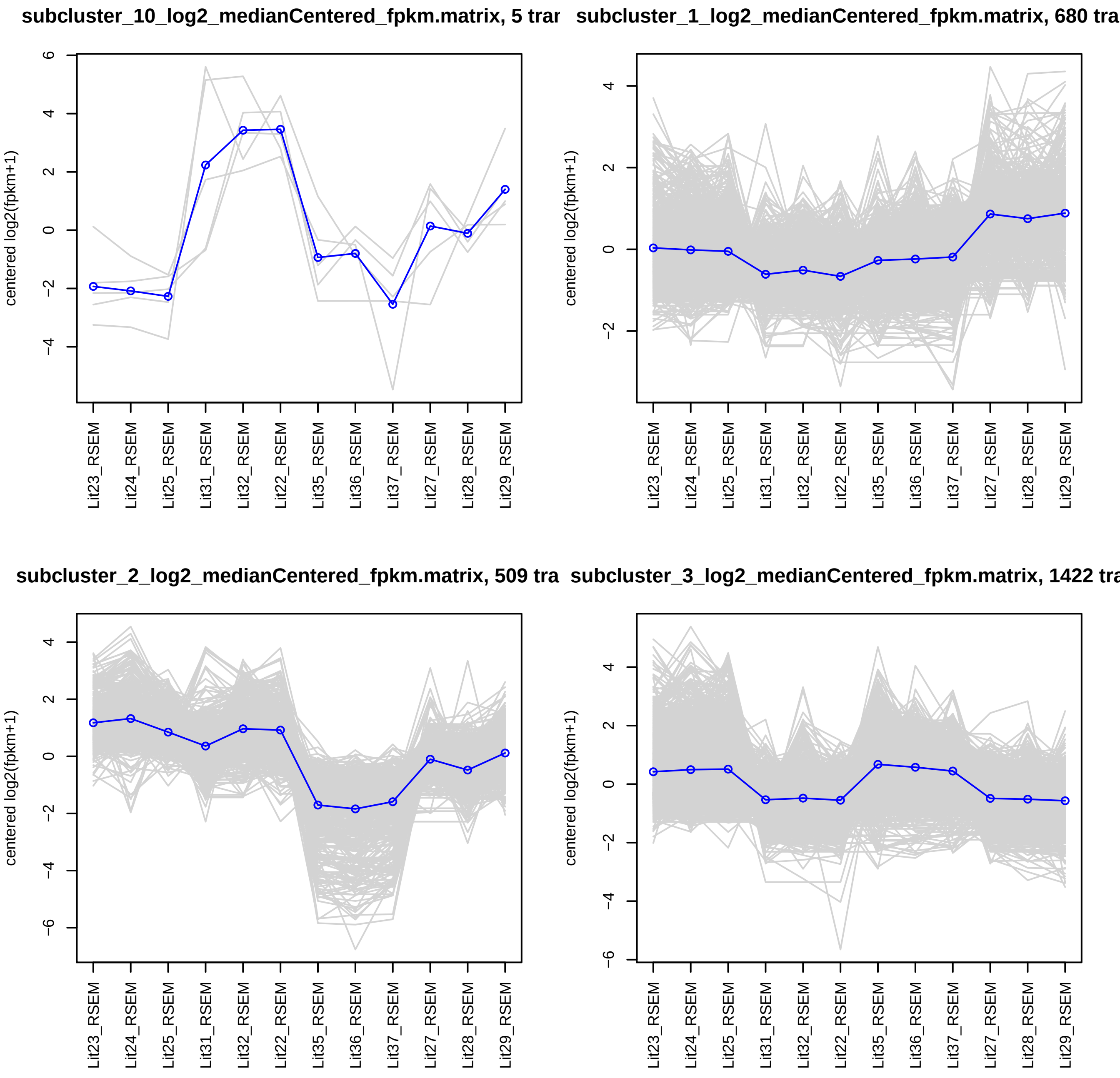
subcluster_10_log2_medianCentered_fpkm.matrix, 5 trar
subcluster_1_log2_medianCentered_fpkm.matrix, 680 tra
subcluster_2_log2_medianCentered_fpkm.matrix, 509 tra
subcluster_3_log2_medianCentered_fpkm.matrix, 1422 tra
centered log2(fpkm+1)
Lit23_RSEM
Lit24_RSEM
Lit25_RSEM
Lit31_RSEM
Lit32_RSEM
Lit22_RSEM
Lit35_RSEM
Lit36_RSEM
Lit37_RSEM
Lit27_RSEM
Lit28_RSEM
Lit29_RSEM

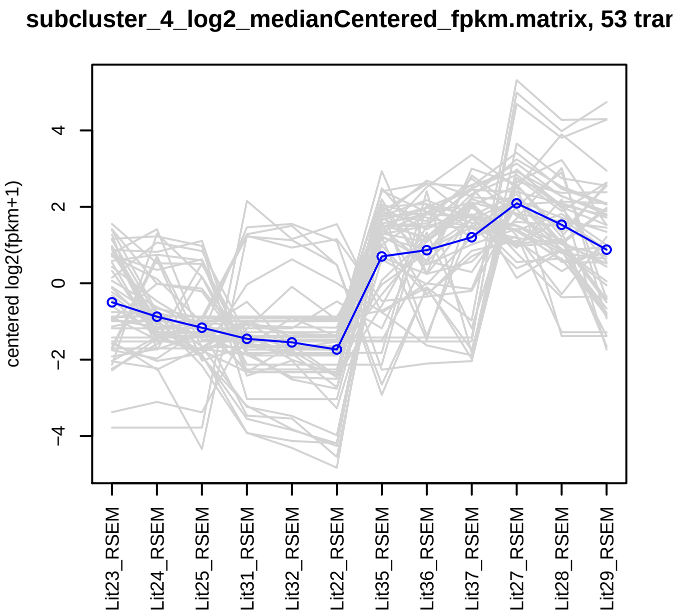
subcluster_4_log2_medianCentered_fpkm.matrix, 53 trar
centered log2(fpkm+1)
Lit23_RSEM
Lit24_RSEM
Lit25_RSEM
Lit31_RSEM
Lit32_RSEM
Lit22_RSEM
Lit35_RSEM
Lit36_RSEM
Lit37_RSEM
Lit27_RSEM
Lit28_RSEM
Lit29_RSEM

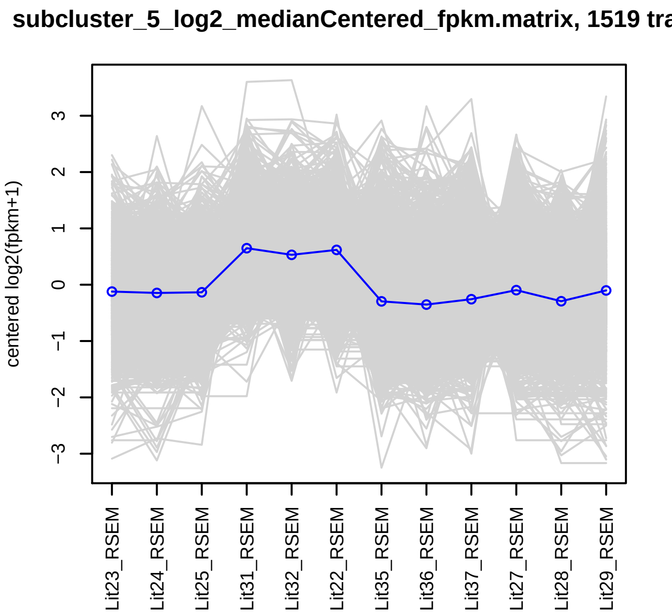
subcluster_5_log2_medianCentered_fpkm.matrix, 1519 tra
centered log2(fpkm+1)
Lit23_RSEM
Lit24_RSEM
Lit25_RSEM
Lit31_RSEM
Lit32_RSEM
Lit22_RSEM
Lit35_RSEM
Lit36_RSEM
Lit37_RSEM
Lit27_RSEM
Lit28_RSEM
Lit29_RSEM

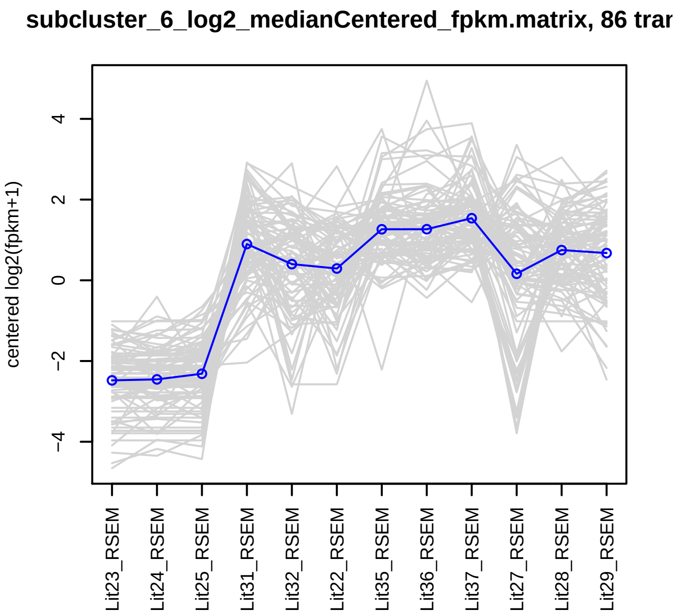
subcluster_6_log2_medianCentered_fpkm.matrix, 86 trar
centered log2(fpkm+1)
Lit23_RSEM
Lit24_RSEM
Lit25_RSEM
Lit31_RSEM
Lit32_RSEM
Lit22_RSEM
Lit35_RSEM
Lit36_RSEM
Lit37_RSEM
Lit27_RSEM
Lit28_RSEM
Lit29_RSEM

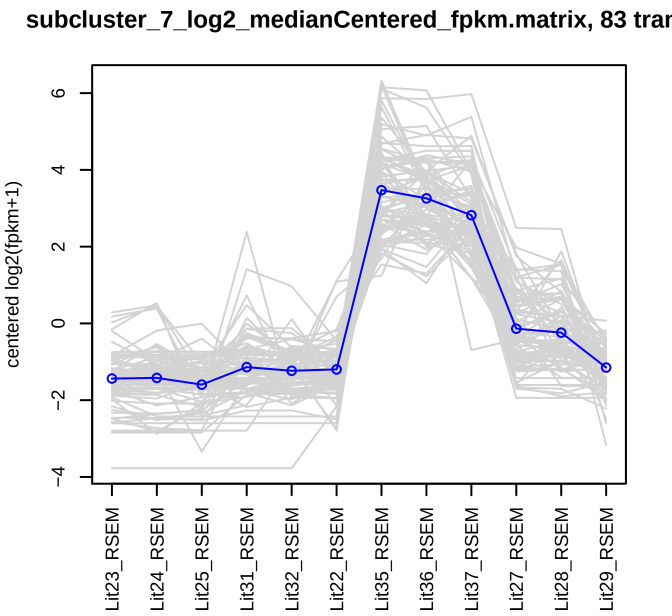
subcluster_7_log2_medianCentered_fpkm.matrix, 83 trar
centered log2(fpkm+1)
Lit23_RSEM
Lit24_RSEM
Lit25_RSEM
Lit31_RSEM
Lit32_RSEM
Lit22_RSEM
Lit35_RSEM
Lit36_RSEM
Lit37_RSEM
Lit27_RSEM
Lit28_RSEM
Lit29_RSEM

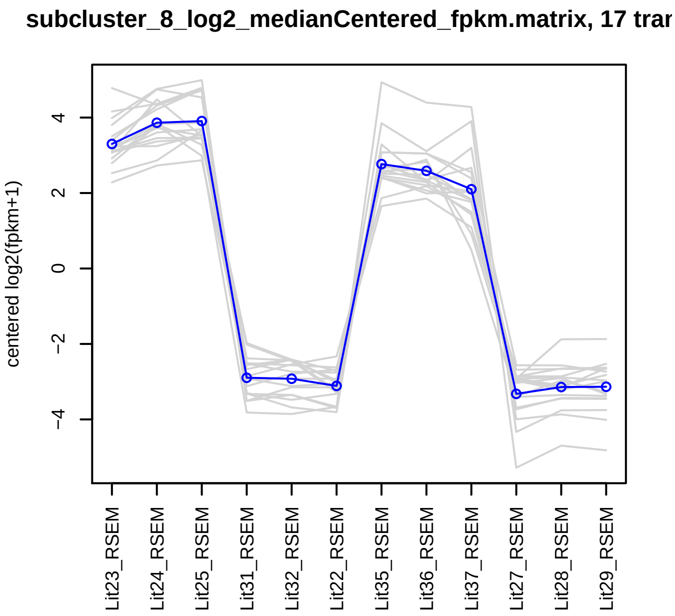
subcluster_8_log2_medianCentered_fpkm.matrix, 17 trar
centered log2(fpkm+1)
4
2
0
−2
−4
Lit23_RSEM
Lit24_RSEM
Lit25_RSEM
Lit31_RSEM
Lit32_RSEM
Lit22_RSEM
Lit35_RSEM
Lit36_RSEM
Lit37_RSEM
Lit27_RSEM
Lit28_RSEM
Lit29_RSEM

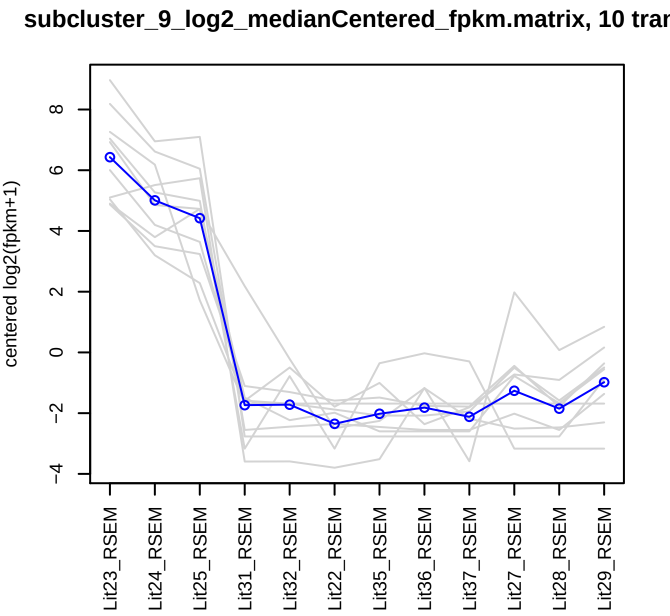
subcluster_9_log2_medianCentered_fpkm.matrix, 10 trar
centered log2(fpkm+1)
8
6
4
2
0
−2
−4
Lit23_RSEM
Lit24_RSEM
Lit25_RSEM
Lit31_RSEM
Lit32_RSEM
Lit22_RSEM
Lit35_RSEM
Lit36_RSEM
Lit37_RSEM
Lit27_RSEM
Lit28_RSEM
Lit29_RSEM
