# Supplementary material for: Family dinner: Transcriptional plasticity of five Noctuidae (Lepidoptera) feeding on three host plant species
Source: Ecol Evol. 2022 Sep 6;12(9):e9258. doi: 10.1002/ece3.9258 (PMC9448971; doi:10.1002/ece3.9258)
Supplement: Supplementary file 52 — Figure S17b [file ECE3-12-e9258-s001.pdf]

subcluster_10_log2_medianCentered_fpkm.matrix, 1 trar

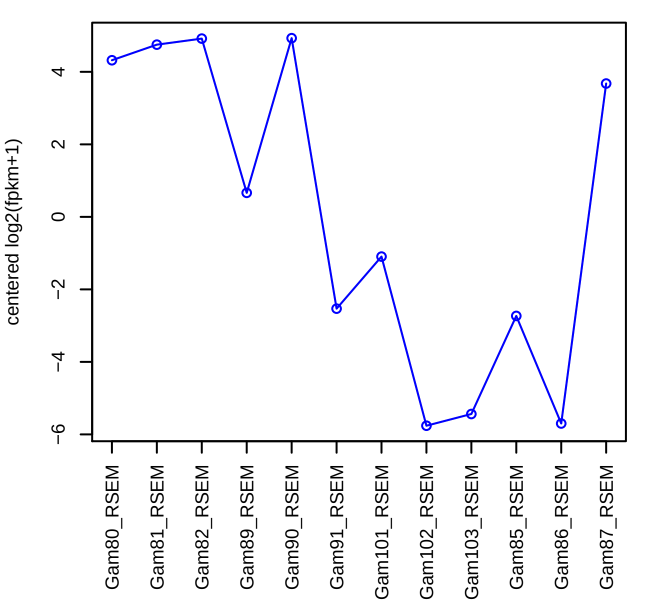

subcluster_11_log2_medianCentered_fpkm.matrix, 2 trar

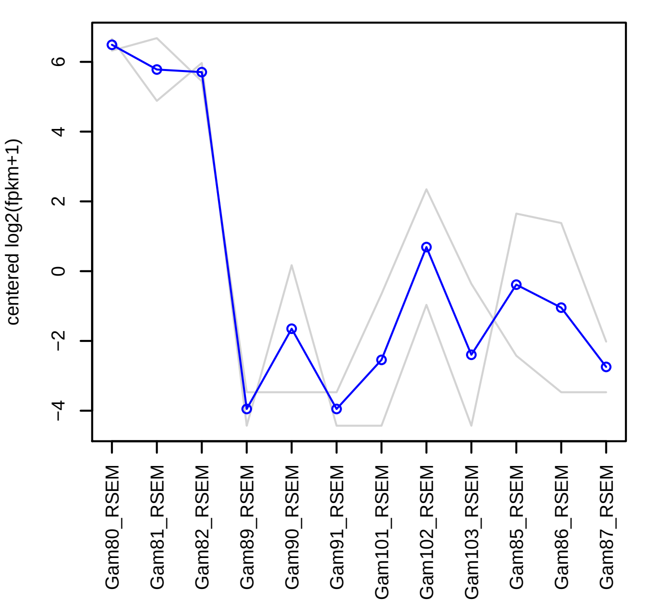

subcluster_12_log2_medianCentered_fpkm.matrix, 5 trar

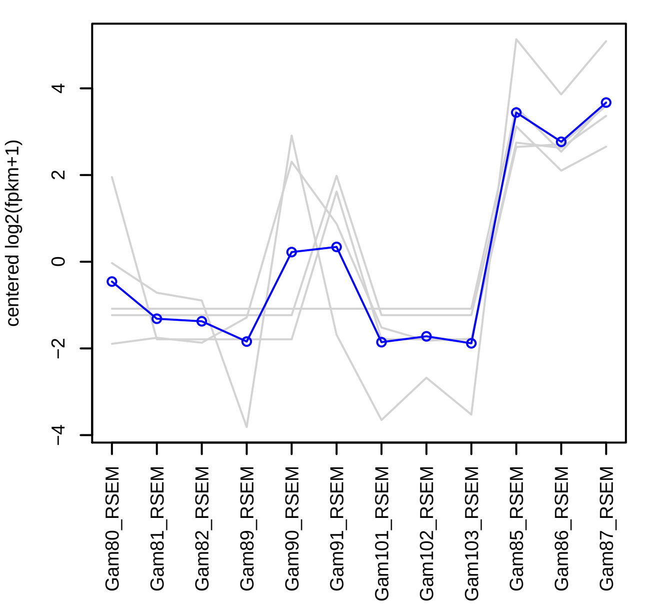

subcluster_13_log2_medianCentered_fpkm.matrix, 7 trar

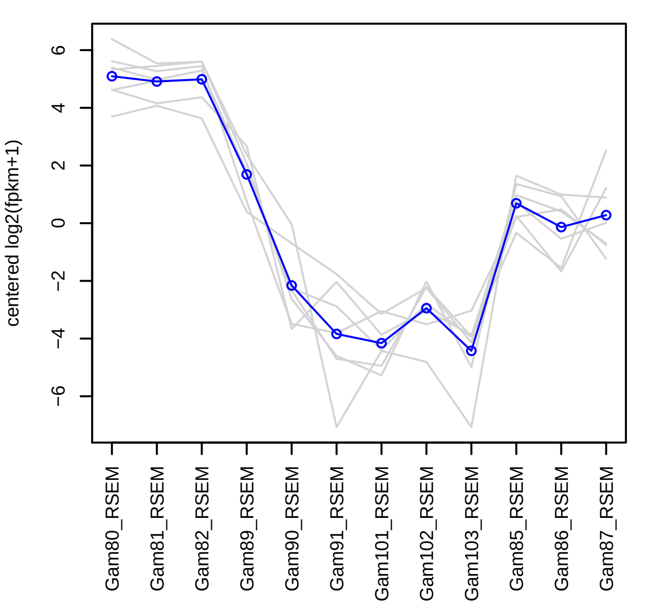

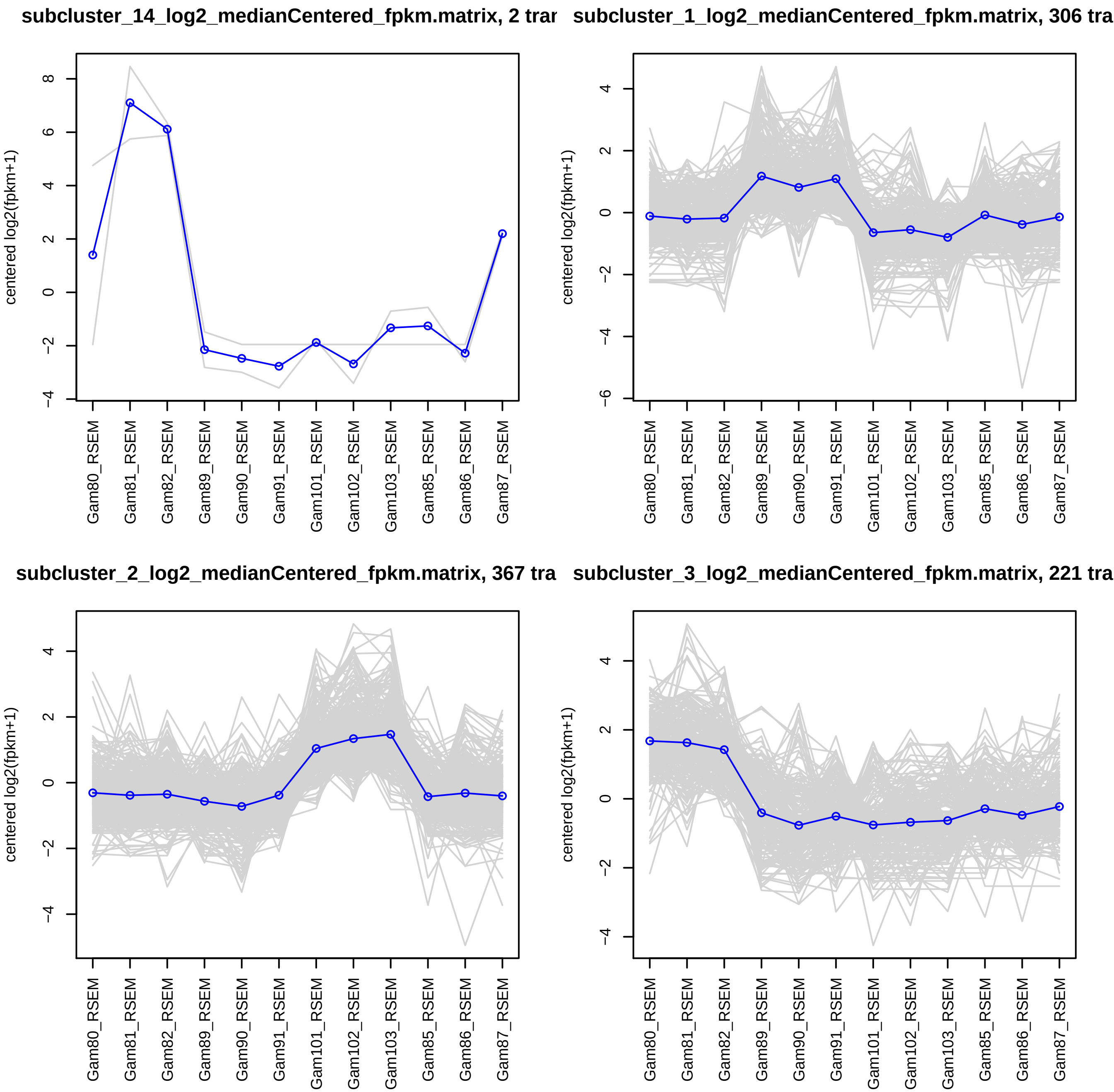
subcluster_14_log2_medianCentered_fpkm.matrix, 2 trar
subcluster_1_log2_medianCentered_fpkm.matrix, 306 tra
subcluster_2_log2_medianCentered_fpkm.matrix, 367 tra
subcluster_3_log2_medianCentered_fpkm.matrix, 221 tra
centered log2(fpkm+1)
Gam80_RSEM
Gam81_RSEM
Gam82_RSEM
Gam89_RSEM
Gam90_RSEM
Gam91_RSEM
Gam101_RSEM
Gam102_RSEM
Gam103_RSEM
Gam85_RSEM
Gam86_RSEM
Gam87_RSEM

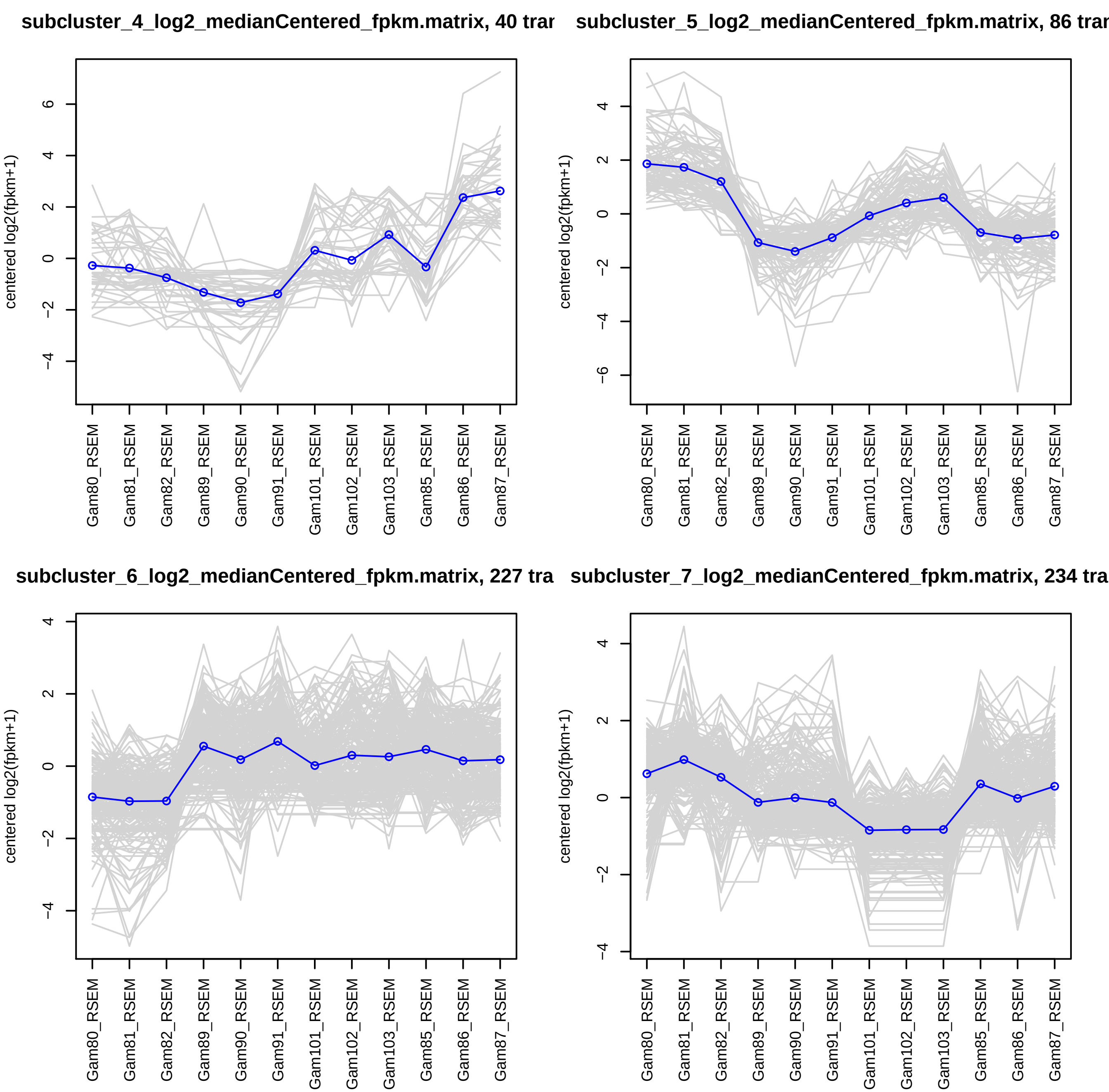
subcluster_4_log2_medianCentered_fpkm.matrix, 40 trar
subcluster_5_log2_medianCentered_fpkm.matrix, 86 trar
subcluster_6_log2_medianCentered_fpkm.matrix, 227 tra
subcluster_7_log2_medianCentered_fpkm.matrix, 234 tra
centered log2(fpkm+1)
Gam80_RSEM
Gam81_RSEM
Gam82_RSEM
Gam89_RSEM
Gam90_RSEM
Gam91_RSEM
Gam101_RSEM
Gam102_RSEM
Gam103_RSEM
Gam85_RSEM
Gam86_RSEM
Gam87_RSEM

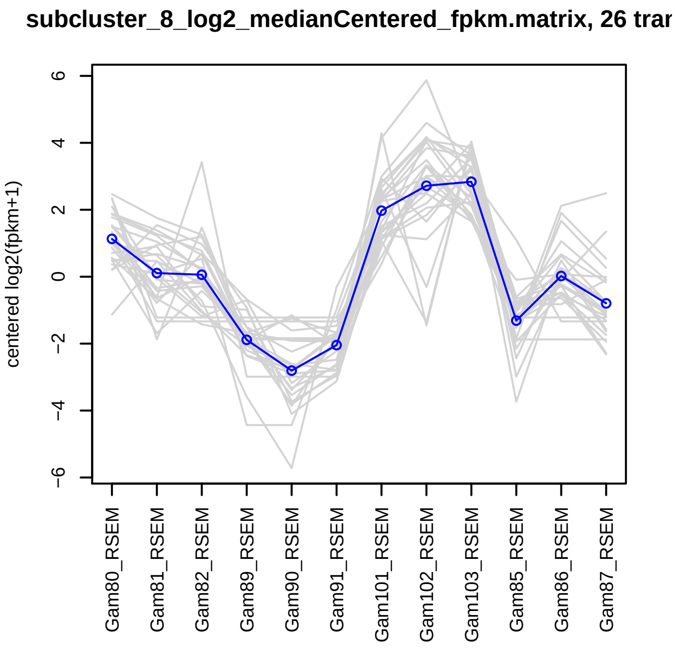
subcluster_8_log2_medianCentered_fpkm.matrix, 26 trar
centered log2(fpkm+1)
6
4
2
0
−2
−4
−6
Gam80_RSEM
Gam81_RSEM
Gam82_RSEM
Gam89_RSEM
Gam90_RSEM
Gam91_RSEM
Gam101_RSEM
Gam102_RSEM
Gam103_RSEM
Gam85_RSEM
Gam86_RSEM
Gam87_RSEM

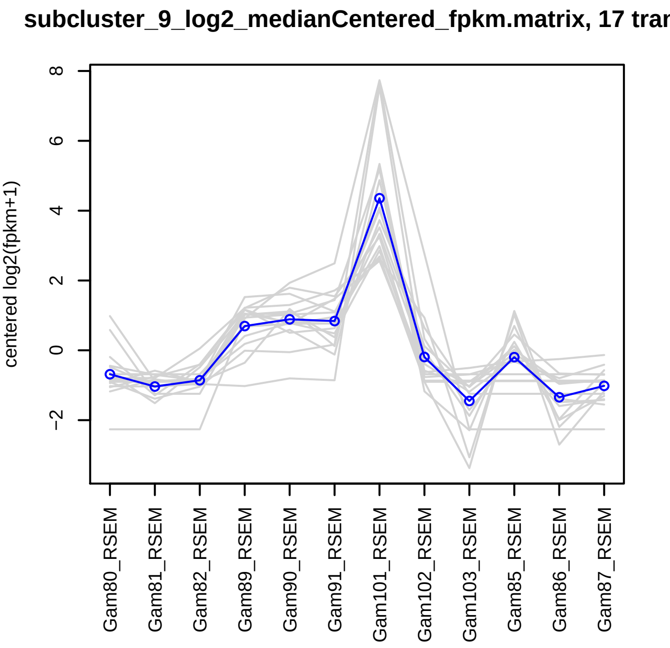
subcluster_9_log2_medianCentered_fpkm.matrix, 17 trar
centered log2(fpkm+1)
8
6
4
2
0
−2
Gam80_RSEM
Gam81_RSEM
Gam82_RSEM
Gam89_RSEM
Gam90_RSEM
Gam91_RSEM
Gam101_RSEM
Gam102_RSEM
Gam103_RSEM
Gam85_RSEM
Gam86_RSEM
Gam87_RSEM
