# Supplementary material for: Family dinner: Transcriptional plasticity of five Noctuidae (Lepidoptera) feeding on three host plant species
Source: Ecol Evol. 2022 Sep 6;12(9):e9258. doi: 10.1002/ece3.9258 (PMC9448971; doi:10.1002/ece3.9258)
Supplement: Supplementary file 53 — Figure S17c [file ECE3-12-e9258-s021.pdf]

subcluster_10_log2_medianCentered_fpkm.matrix, 63 tra

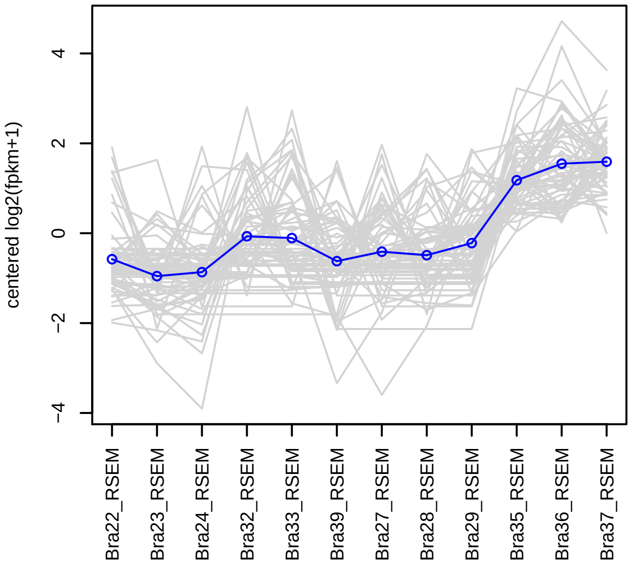

subcluster_11_log2_medianCentered_fpkm.matrix, 5 trar

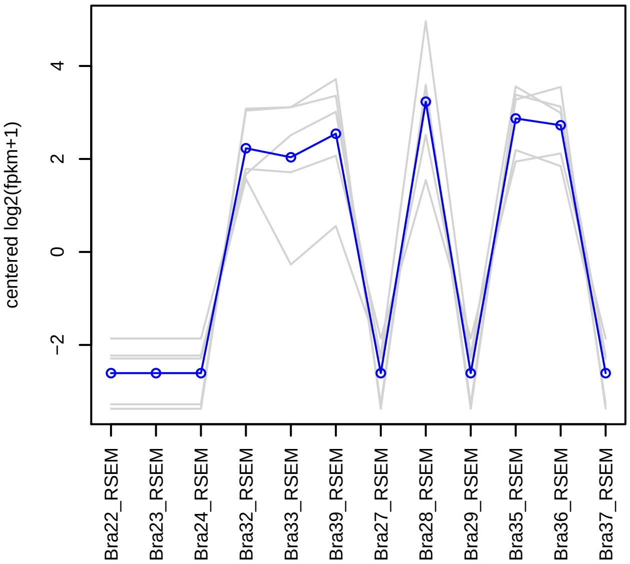

subcluster_12_log2_medianCentered_fpkm.matrix, 14 tra

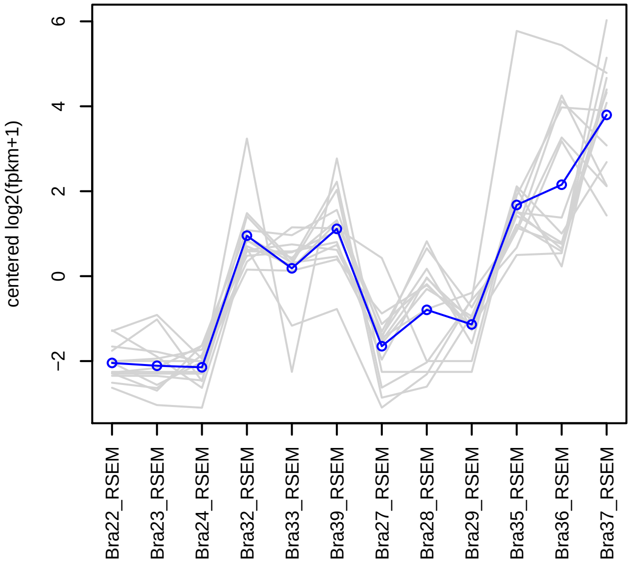

subcluster_1_log2_medianCentered_fpkm.matrix, 439 tra

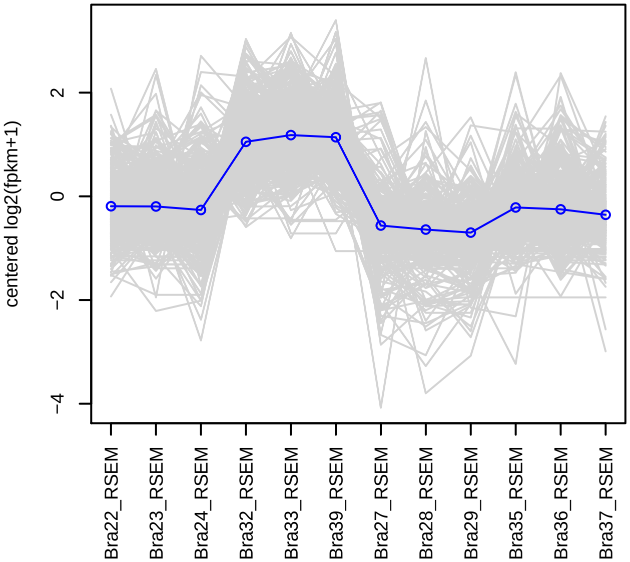

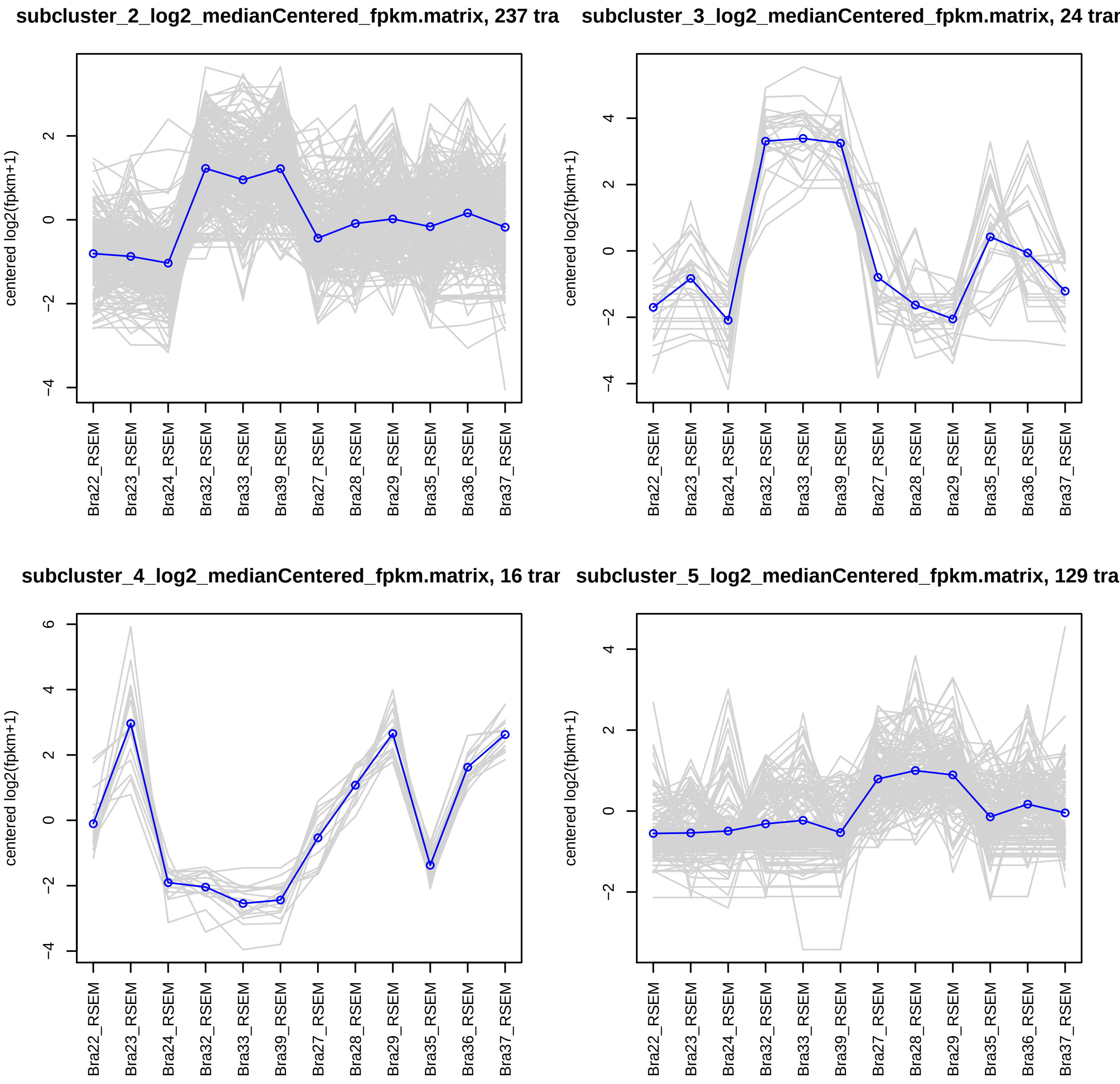
subcluster_2_log2_medianCentered_fpkm.matrix, 237 tra
subcluster_3_log2_medianCentered_fpkm.matrix, 24 trar
subcluster_4_log2_medianCentered_fpkm.matrix, 16 trar
subcluster_5_log2_medianCentered_fpkm.matrix, 129 tra
centered log2(fpkm+1)
Bra22_RSEM
Bra23_RSEM
Bra24_RSEM
Bra32_RSEM
Bra33_RSEM
Bra39_RSEM
Bra27_RSEM
Bra28_RSEM
Bra29_RSEM
Bra35_RSEM
Bra36_RSEM
Bra37_RSEM
−4
−2
0
2
4
6

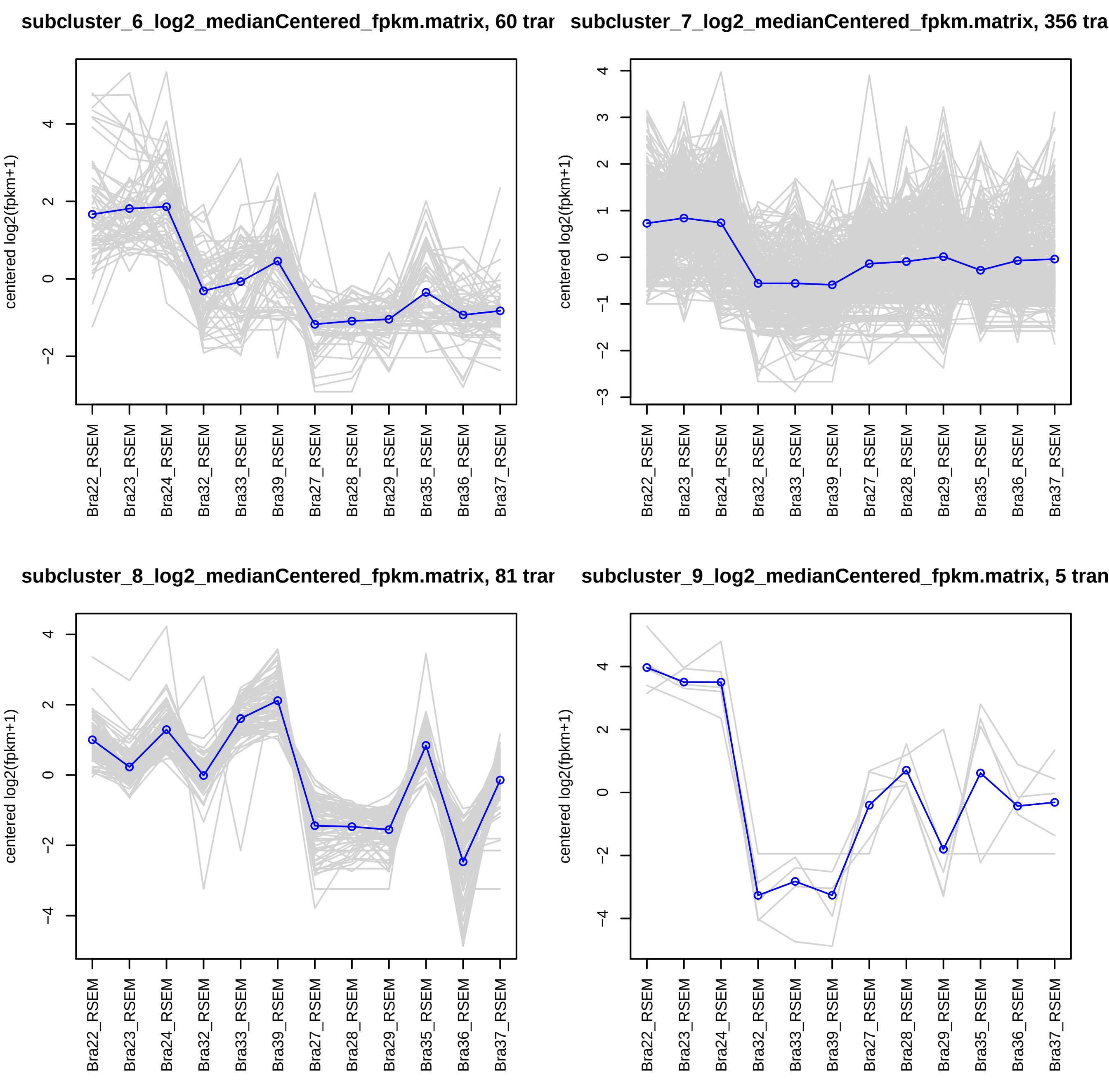

subcluster_6_log2_medianCentered_fpkm.matrix, 60 trar
subcluster_7_log2_medianCentered_fpkm.matrix, 356 tra
subcluster_8_log2_medianCentered_fpkm.matrix, 81 trar
subcluster_9_log2_medianCentered_fpkm.matrix, 5 tran
centered log2(fpkm+1)
Bra22_RSEM
Bra23_RSEM
Bra24_RSEM
Bra32_RSEM
Bra33_RSEM
Bra39_RSEM
Bra27_RSEM
Bra28_RSEM
Bra29_RSEM
Bra35_RSEM
Bra36_RSEM
Bra37_RSEM
