# Supplementary material for: Family dinner: Transcriptional plasticity of five Noctuidae (Lepidoptera) feeding on three host plant species
Source: Ecol Evol. 2022 Sep 6;12(9):e9258. doi: 10.1002/ece3.9258 (PMC9448971; doi:10.1002/ece3.9258)

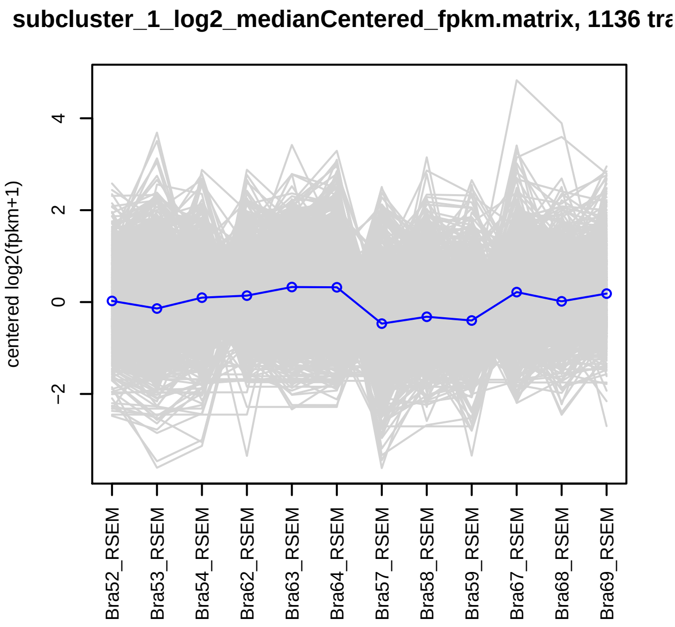
subcluster_1_log2_medianCentered_fpkm.matrix, 1136 tra
centered log2(fpkm+1)
4
2
0
−2
Bra52_RSEM
Bra53_RSEM
Bra54_RSEM
Bra62_RSEM
Bra63_RSEM
Bra64_RSEM
Bra57_RSEM
Bra58_RSEM
Bra59_RSEM
Bra67_RSEM
Bra68_RSEM
Bra69_RSEM

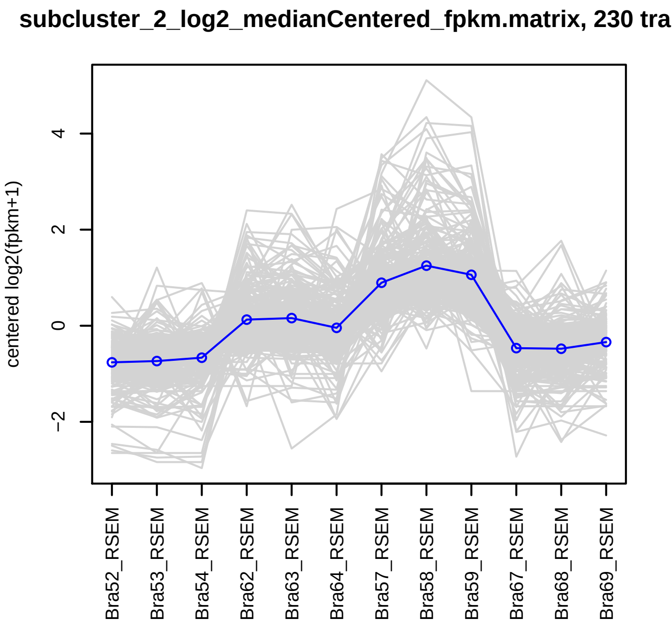
subcluster_2_log2_medianCentered_fpkm.matrix, 230 tra
centered log2(fpkm+1)
4
2
0
−2
Bra52_RSEM
Bra53_RSEM
Bra54_RSEM
Bra62_RSEM
Bra63_RSEM
Bra64_RSEM
Bra57_RSEM
Bra58_RSEM
Bra59_RSEM
Bra67_RSEM
Bra68_RSEM
Bra69_RSEM

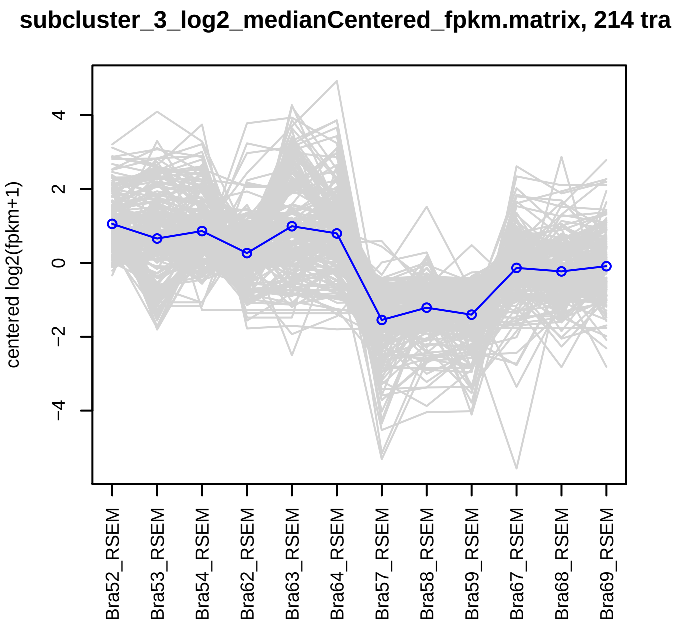
subcluster_3_log2_medianCentered_fpkm.matrix, 214 tra
centered log2(fpkm+1)
4
2
0
−2
−4
Bra52_RSEM
Bra53_RSEM
Bra54_RSEM
Bra62_RSEM
Bra63_RSEM
Bra64_RSEM
Bra57_RSEM
Bra58_RSEM
Bra59_RSEM
Bra67_RSEM
Bra68_RSEM
Bra69_RSEM

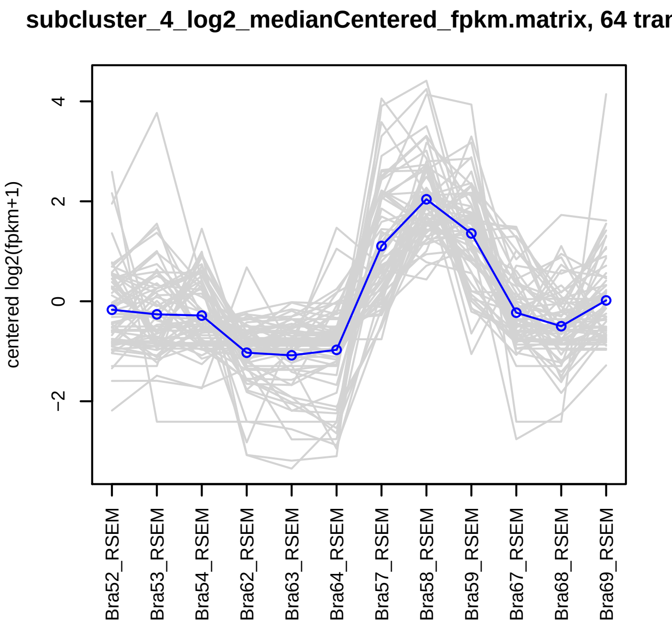
subcluster_4_log2_medianCentered_fpkm.matrix, 64 trar
centered log2(fpkm+1)
4
2
0
−2
Bra52_RSEM
Bra53_RSEM
Bra54_RSEM
Bra62_RSEM
Bra63_RSEM
Bra64_RSEM
Bra57_RSEM
Bra58_RSEM
Bra59_RSEM
Bra67_RSEM
Bra68_RSEM
Bra69_RSEM

subcluster_5_log2_medianCentered_fpkm.matrix, 23 trar

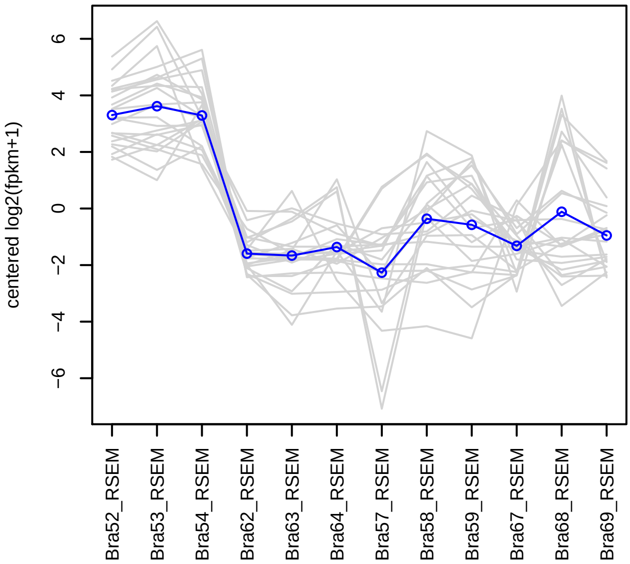

subcluster_6_log2_medianCentered_fpkm.matrix, 62 trar

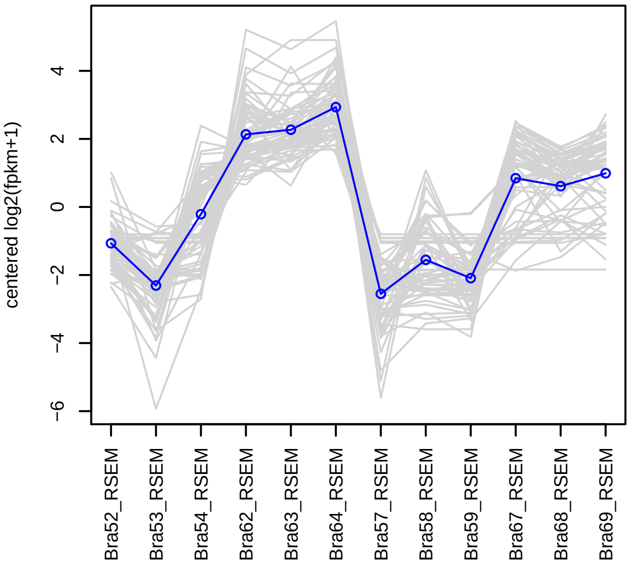

subcluster_7_log2_medianCentered_fpkm.matrix, 1 tran

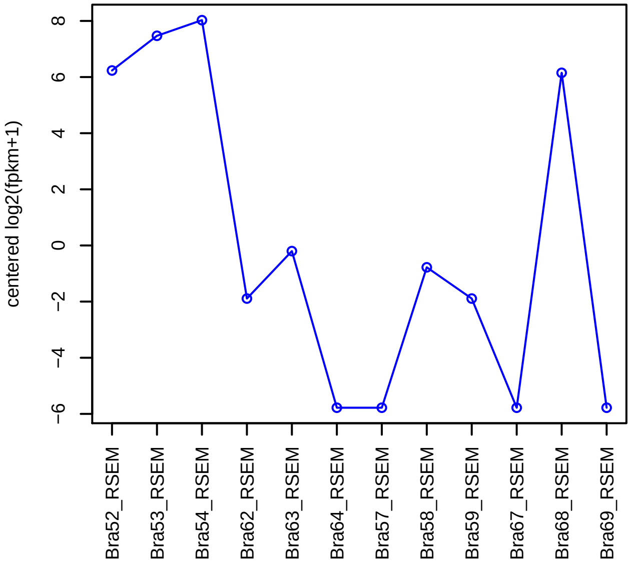

subcluster_8_log2_medianCentered_fpkm.matrix, 4 tran

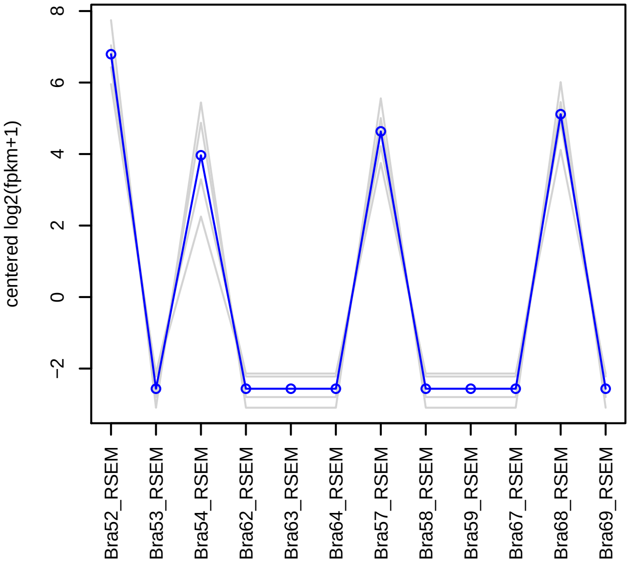

subcluster_9_log2_medianCentered_fpkm.matrix, 1 tran

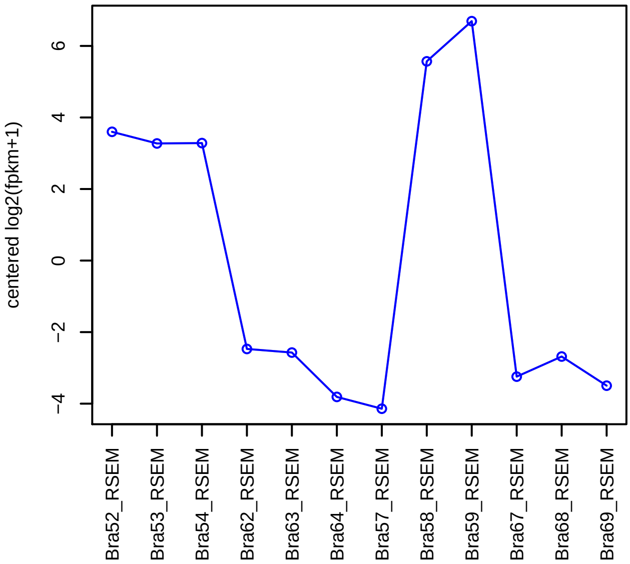

Supplement: Supplementary file 54 — Figure S17d [file ECE3-12-e9258-s037.pdf]
