# Supplementary material for: Family dinner: Transcriptional plasticity of five Noctuidae (Lepidoptera) feeding on three host plant species
Source: Ecol Evol. 2022 Sep 6;12(9):e9258. doi: 10.1002/ece3.9258 (PMC9448971; doi:10.1002/ece3.9258)
Supplement: Supplementary file 60 — File S23 [file ECE3-12-e9258-s024.docx]

**Supplementary data file 23**

**Supplementary Results and Discussion**

***Brassica oleracea***

In *B. oleracea* chewing insects activate the production of bioactive compounds, such as isothiocyanates (ICTs), by hydrolyses of glucosinolates due to release of myrosinases to glucosinolates during chewing [1]. General detoxification enzymes like GST’s have been found to detoxify ITCs like GST epsilon 1 (GSTE1) that was upregulated in *S. litura* when feeding on glucosinolate-rich Cruciferae [2]. GSTE1 was found DE in both *S. exigua* and *M. brassicae* in this study*.* GSTE1 was clustered in cluster 2 for *S. exigua,* upregulated in all samples of the artificial diet and only in larvae of a single sample feeding on *B. oleracea.* Yet, in *M. brassicae,* GSTE1 was only upregulated in all *B. oleracea* diet treatments indicating that this gene is potentially involved in glucosinolate detoxification in *M. brassicae*.

All species showed to have genes clustered in diet specific expression clusters showing upregulation for *B. oleracea*. The Noctuidae showed overlap in expressed gene clans and subgroups within the various detoxification gene families. The amount of differently expressed and upregulated detoxification genes did differ between the species (Supplementary table 6). Both *S. littoralis* and *M. brassicae* showed for several gene families a higher number of genes upregulated for *B. oleracea* than for the other diet treatments, while this was restricted to UGT members for *A. gamma.* Further, *S. exigua* was lacking any upregulated P450 gene family members and *T. ni* had no upregulated CCE genes. For P450 primarily clan-3 genes are upregulated, for UGT most genes are identified as UGT33 and UGT40, for GST many genes are expressed from the microsomal, delta and omega groups, for CCE most genes are from the 1 and 16 subgroups and for ABC most genes are ABC-A, ABC-B and ABC-G.

***Zea mays***

For *Z. mays* only *A. gamma*, *M. brassicae* and *S. littoralis* showed a host-specific expression cluster with upregulated genes in larvae feeding on this host plant species. Both *T. ni* and *S. exigua* [3] had no specific *Z. mays* upregulated expression clusters. Detoxification genes were only upregulated in the larvae of *M. brassicae* and *S. littoralis* feeding on this host plant*.*.

The main specialized metabolite employed by *Z. mays* are benzoxazinoids [4]. Glucosylation has been found to be the main metabolism strategy of *Spodoptera* species against benzoxazinoids, which potentially involves employing UGT genes for detoxification [5, 6]. Among the UGTs annotated in *S. littoralis* is UGT40. Members of this group are often found expressed in digestion and detoxification-related body parts in related Lepidoptera species [7] and has been found expressed in the fat body of *S. exigua* larvae [8] and upregulated in *N. tabacum* fed larvae [3]. This indicates that UGT40 is most likely involved in detoxification and digestion in general but is not *Z. mays* specific in *Spodoptera.* Members of other detoxification families then UGT were also identified in *S. littoralis* larvae feeding on *Z. mays* (Supplementary table 6).  All these differentially expressed genes, clustered in cluster 1 (Figure 2), represented all 5 major detoxification gene families. Of the identified P450s, 7 are CYP6 members which are part of the large clan-3 P450s. Further, 9 are part of the clan-4 P450s all identified as CYP4 members and a single gene is identified as CYP304, member of clan-2. The largest clan in insect P450s is clan-3, well known to be involved in detoxification of plant defence toxins and insecticide resistance [e.g. 9, 10, 11]. Clan-4 P450s are numerous in insects too and do show a great diversity in functions from pheromone to xenobiotics metabolism, while clan-2 P450s mainly have important physiological roles [12].

Further, a variety of ABC gene families have been found expressed in cluster 1 including members belonging to the ABC-A, ABC-C, ABC-D and ABC-G groups. Genes from the ABC-B, ABC-C, ABC-G and ABC-H subfamilies are known to be involved in xenobiotic metabolism within Lepidoptera while for the other groups genes are involved in general transportation and other regular processes [13-16]. In phloem- and blood-sucking arthropods an expansion of the subfamilies ABC-G and ABC-H are seen when compared to other species. Moreover, genes from these subfamilies show overexpression in response to xenobiotics in Lepidoptera species [16, 17].

A limited number of detoxification genes are annotated in the *Z. mays* specific expression cluster of *M. brassicae*: only 4 members of UGT, 3 oxidoreductases, 2 GSTs, and a single ABC gene are upregulated in expression cluster 10 (Figure 2). All 4 UGTs are identified to belong to the UGT33 family which has expanded within Lepidoptera [18, 19] and are often involved in detoxification/digestion of plant material [7, 20, 21]. Of the 2 GST genes only one is identified further and belongs to the epsilon class. The epsilon and delta classes of the in total 6 classes within GST are only found in Arthropoda and often associated with insecticide resistance [e.g. 22, 23].

***Nicotiana tabacum***

For *N. tabacum* all species showed to have genes clustered in a diet specific expression cluster showing upregulation for the particular host plant species (Figure 2). However, *S. littoralis* was lacking any P450s and GST genes in the cluster showing upregulation for *N. tabacum* samples (Supplementary table 6). This suggests that *S. littoralis* for the detoxification of alkaloids potentially relies on the other detoxification gene families that were differentially expressed: CCE, UGT, ABC, GSS and GMC. The other species upregulated P450s from the detoxification-related clan-3: CYP9s, CYP6s and CYP321. CYP321 was only expressed by *T. ni* and *A. gamma* and has been confirmed to play a role in xenobiotic detoxification [24, 25]. Further, *M. brassicae* was lacking any upregulated UGT and ABC gene family members in larvae feeding on *N. tabacum*. Further, all species showed upregulation of CCE members in larvae feeding on leaf material of which *T. ni* and *A. gamma* (both Plusiinae members) showing the highest number expressed compared to the others. Expansions of the CCE gene family are found in generalist Noctuidae and their role in detoxification of xenobiotics has been shown of importance [7, 26, 27]. The results found here could indicate that CCEs are of greater importance for Plusiinae in alkaloid detoxification present in *N. tabacum* compared to the Noctuinae species included in this study.

**References**

1. Kliebenstein DJ, Kroymann J, Mitchell-Olds T: **The glucosinolate–myrosinase system in an ecological and evolutionary context**. *Current opinion in plant biology* 2005, **8**(3):264-271.

2. Zou X, Xu Z, Zou H, Liu J, Chen S, Feng Q, Zheng S: **Glutathione S-transferase SlGSTE1 in Spodoptera litura may be associated with feeding adaptation of host plants**. *Insect biochemistry and molecular biology* 2016, **70**:32-43.

3. Breeschoten T, Ros VI, Schranz ME, Simon S: **An influential meal: host plant dependent transcriptional variation in the beet armyworm, Spodoptera exigua (Lepidoptera: Noctuidae)**. *BMC genomics* 2019, **20**(1):1-15.

4. Wouters FC, Blanchette B, Gershenzon J, Vassão DG: **Plant defense and herbivore counter-defense: benzoxazinoids and insect herbivores**. *Phytochemistry Reviews* 2016, **15**(6):1127-1151.

5. Wouters FC, Reichelt M, Glauser G, Bauer E, Erb M, Gershenzon J, Vassão DG: **Reglucosylation of the benzoxazinoid DIMBOA with inversion of stereochemical configuration is a detoxification strategy in lepidopteran herbivores**. *Angewandte Chemie* 2014, **126**(42):11502-11506.

6. Roy A, Walker III W, Vogel H, Chattington S, Larsson M, Anderson P, Heckel DG, Schlyter F: **Diet dependent metabolic responses in three generalist insect herbivores Spodoptera spp**. *Insect biochemistry and molecular biology* 2016, **71**:91-105.

7. Pearce SL, Clarke DF, East PD, Elfekih S, Gordon K, Jermiin LS, McGaughran A, Oakeshott JG, Papanikolaou A, Perera OP: **Genomic innovations, transcriptional plasticity and gene loss underlying the evolution and divergence of two highly polyphagous and invasive Helicoverpa pest species**. *BMC biology* 2017, **15**(1):63.

8. Hu B, Zhang SH, Ren MM, Tian XR, Wei Q, Mburu DK, Su JY: **The expression of Spodoptera exigua P450 and UGT genes: tissue specificity and response to insecticides**. *Insect science* 2019, **26**(2):199-216.

9. Chandra GS, Asokan R, Manamohan M, Sita T: **Cytochrome P450 isoforms transcriptional, larval growth and development responses to host allelochemicals in the generalist herbivore, Helicoverpa armigera (Hubner)(Lepidoptera: Noctuidae)**. *Current Science* 2016, **111**(5):901-906.

10. Zhu F, Liu N: **Differential expression of CYP6A5 and CYP6A5v2 in pyrethroid‐resistant house flies, Musca domestica**. *Archives of insect biochemistry and physiology* 2008, **67**(3):107-119.

11. Wen Z, Rupasinghe S, Niu G, Berenbaum MR, Schuler MA: **CYP6B1 and CYP6B3 of the black swallowtail (Papilio polyxenes): adaptive evolution through subfunctionalization**. *Molecular biology and evolution* 2006, **23**(12):2434-2443.

12. Feyereisen R: **Evolution of insect P450**. In*.*: Portland Press Limited; 2006.

13. Saurin W, Hofnung M, Dassa E: **Getting in or out: early segregation between importers and exporters in the evolution of ATP-binding cassette (ABC) transporters**. *Journal of molecular evolution* 1999, **48**(1):22-41.

14. Liu S, Zhou S, Tian L, Guo E, Luan Y, Zhang J, Li S: **Genome-wide identification and characterization of ATP-binding cassette transporters in the silkworm, Bombyx mori**. *BMC genomics* 2011, **12**(1):491.

15. Labbé R, Caveney S, Donly C: **Genetic analysis of the xenobiotic resistance‐associated ABC gene subfamilies of the Lepidoptera**. *Insect molecular biology* 2011, **20**(2):243-256.

16. Bretschneider A, Heckel DG, Vogel H: **Know your ABCs: Characterization and gene expression dynamics of ABC transporters in the polyphagous herbivore Helicoverpa armigera**. *Insect biochemistry and molecular biology* 2016, **72**:1-9.

17. Tian L, Song T, He R, Zeng Y, Xie W, Wu Q, Wang S, Zhou X, Zhang Y: **Genome-wide analysis of ATP-binding cassette (ABC) transporters in the sweetpotato whitefly, Bemisia tabaci**. *BMC genomics* 2017, **18**(1):330.

18. Gouin A, Bretaudeau A, Nam K, Gimenez S, Aury J-M, Duvic B, Hilliou F, Durand N, Montagné N, Darboux I: **Two genomes of highly polyphagous lepidopteran pests (Spodoptera frugiperda, Noctuidae) with different host-plant ranges**. *Scientific Reports* 2017, **7**(1):11816.

19. Ahn S-J, Vogel H, Heckel DG: **Comparative analysis of the UDP-glycosyltransferase multigene family in insects**. *Insect biochemistry and molecular biology* 2012, **42**(2):133-147.

20. Bock KW: **The UDP-glycosyltransferase (UGT) superfamily expressed in humans, insects and plants: Animal⿿ plant arms-race and co-evolution**. *Biochemical pharmacology* 2016, **99**:11-17.

21. Schweizer F, Heidel-Fischer H, Vogel H, Reymond P: **Arabidopsis glucosinolates trigger a contrasting transcriptomic response in a generalist and a specialist herbivore**. *Insect Biochemistry and Molecular Biology* 2017, **85**:21-31.

22. Deng H, Huang Y, Feng Q, Zheng S: **Two epsilon glutathione S-transferase cDNAs from the common cutworm, Spodoptera litura: Characterization and developmental and induced expression by insecticides**. *Journal of insect physiology* 2009, **55**(12):1174-1183.

23. Lumjuan N, Rajatileka S, Changsom D, Wicheer J, Leelapat P, Prapanthadara L-a, Somboon P, Lycett G, Ranson H: **The role of the Aedes aegypti Epsilon glutathione transferases in conferring resistance to DDT and pyrethroid insecticides**. *Insect biochemistry and molecular biology* 2011, **41**(3):203-209.

24. Wang R-L, He Y-N, Staehelin C, Liu S-W, Su Y-J, Zhang J-E: **Identification of two cytochrome monooxygenase P450 genes, CYP321A7 and CYP321A9, from the tobacco cutworm moth (Spodoptera litura) and their expression in response to plant allelochemicals**. *International journal of molecular sciences* 2017, **18**(11):2278.

25. Wang RL, Zhu‐Salzman K, Baerson SR, Xin XW, Li J, Su YJ, Zeng RS: **Identification of a novel cytochrome P450 CYP321B1 gene from tobacco cutworm (Spodoptera litura) and RNA interference to evaluate its role in commonly used insecticides**. *Insect science* 2017, **24**(2):235-247.

26. Cheng T, Wu J, Wu Y, Chilukuri RV, Huang L, Yamamoto K, Feng L, Li W, Chen Z, Guo H: **Genomic adaptation to polyphagy and insecticides in a major East Asian noctuid pest**. *Nature ecology & evolution* 2017, **1**(11):1747.

27. Oakeshott J, Claudianos C, Campbell P, Newcomb R, Russell R, Gilbert L: **Biochemical genetics and genomics of insect esterases**. *Comprehensive molecular insect science Volume* 2005, **5**.
